# Topoisomerase 3b facilitates piRNA biogenesis to promote transposon silencing and germ cell development

**DOI:** 10.1016/j.celrep.2025.115495

**Published:** 2025-04-03

**Authors:** Seung Kyu Lee, Weiping Shen, William Wen, Yuyoung Joo, Yutong Xue, Aaron Park, Amy Qiang, Shuaikun Su, Tianyi Zhang, Megan Zhang, Jinshui Fan, Yongqing Zhang, Supriyo De, Ildar Gainetdinov, Alexei Sharov, Manolis Maragkakis, Weidong Wang

**Affiliations:** 1 Laboratory of Genetics and Genomics, National Institute on Aging, National Institutes of Health, Baltimore, MD 21224, USA; 2 Department of Biology, New York University, New York, NY 10003, USA; 3 Lead contact

## Abstract

Topoisomerases typically function in the nucleus to relieve topological stress in DNA. Here, we show that a dual-activity topoisomerase, Top3b, and its partner, TDRD3, largely localize in the cytoplasm and interact biochemically and genetically with PIWI-interacting RNA (piRNA) processing enzymes to promote piRNA biogenesis, post-transcriptional gene silencing (PTGS) of transposons, and *Drosophila* germ cell development. Top3b requires its topoisomerase activity to promote PTGS of a transposon reporter and preferentially silences long and highly expressed transposons, suggesting that RNAs with these features may produce more topological stress for topoisomerases to solve. The double mutants between *Top3b* and piRNA processing enzymes exhibit stronger disruption of the signatures and levels of germline piRNAs, more desilenced transposons, and larger defects in germ cells than either single mutant. Our data suggest that Top3b can act in an RNA-based process—piRNA biogenesis and PTGS of transposons—and this function is required for Top3b to promote normal germ cell function.

## In brief

Topoisomerases typically function in the nucleus to solve DNA topological problems. Lee et al. demonstrated that topoisomerase 3b mainly localizes in the cytoplasm and works with piRNA processing enzymes to promote piRNA biogenesis, post-transcriptional silencing of transposons, and germ cell development. The data suggest that topoisomerases may solve topological problems in RNA.

## Graphical Abstract

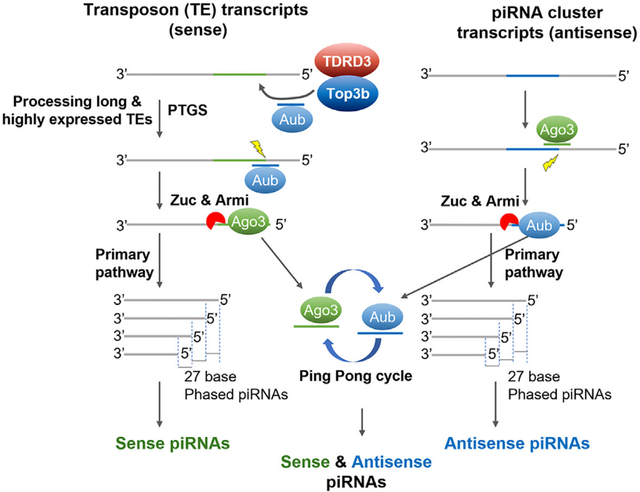

## INTRODUCTION

Topoisomerases typically function within the nucleus to release topological stress produced during DNA replication, transcription, and chromosome segregation.^1,2^ Mutation or dysregulation of topoisomerases can lead to shortened lifespan, cancer, neurological disorders, and lethality.^3–7^ One challenging question regarding topoisomerases is whether they also function in RNA-based cellular processes. We have previously reported that a type IA topoisomerase from humans, Top3b (topoisomerase 3b), is a dual-activity topoisomerase that can change the topology of both DNA and RNA.^8–11^ In addition, this dual activity is prevalent in type IA topoisomerases from all domains of life,^9^ suggesting that RNA metabolism may produce topological problems that depend on Top3b and other type IA topoisomerases to solve.^9,12^ In support of this, Top3b localizes in both nucleus and cytoplasm^10^ and functions in cellular processes on both DNA^13,14^ and mRNA.^14^

Increasing evidence shows that Top3b and its partner TDRD3 act as a complex that participates in three RNA-based processes. First, Top3b-TDRD3 stably associates with RNA binding proteins (RBPs) and translation machinery, including FMRP (fragile X mental retardation syndrome protein), to regulate translation and turnover of mRNAs.^8,10,11,14,15^ Second, Top3b-TDRD3 interacts with the *Drosophila* RNA-induced silencing complex (RISC) to facilitate small interfering RNA (siRNA)-guided heterochromatin formation and transcriptional gene silencing (TGS) of transposable elements (TEs).^16^ However, the role of RISC in heterochromatin formation is controversial, as conflicting evidence has been reported that AGO2, a crucial component of RISC, is required^17–21^ or dispensable^22^ for this process. Third, Top3b-TDRD3 has been reported to be required for replication of several positive-strand RNA viruses, including human coronavirus, SARS-CoV-2 (though not mouse coronavirus, MHV).^23,24^

Mutations of both *Top3b*^10,13,15,25,26^ and *TDRD3*^27–30^ have been linked to neurological and behavior disorders in human, mouse,^13,31^ and *Drosophila*.^8,10^ Interestingly, *Top3b* and *TDRD3* mutations have also been linked to germ cell dysfunction, including age at natural menopause.^32–35^ Moreover, *TDRD3* variants are associated with premature menopause^36^ and polycystic ovary syndrome.^37^ Furthermore, human individuals and mice carrying *Top3b* mutations show defective spermatogenesis and infertility.^38,39^ These data indicate that Top3b-TDRD3 may function critically in germ cells in mammals. However, the underlying mechanism remains unclear.

Here, we show that, unlike other topoisomerases that act in DNA-based processes in the nucleus, Top3b-TDRD3 primarily localizes and functions in the cytoplasm of *Drosophila* germ cells and interacts with PIWI-interacting RNA (piRNA) processing enzymes to promote piRNA biogenesis, post-transcriptional gene silencing (PTGS) of TEs, and germ cell development. Top3b binds TE-derived transcripts, preferentially silences those that are both long and highly expressed, and enhances biogenesis of sense piRNAs that are derived from TE transcripts. The double mutants between *Top3b* and piRNA processing enzymes exhibit stronger disruption of piRNA signatures of both primary and ping-pong pathways, bigger decreases in levels of germline piRNAs, more de-silenced transposons, and larger defects in germ cells than either single mutant. Our data reveal a role of topoisomerases in RNA-based processes—piRNA biogenesis and PTGS of TEs—and suggest a mechanism explaining how *Top3b-TDRD3* mutations can lead to germ cell dysfunction.

## RESULTS

### Top3b-TDRD3 largely localizes in the cytoplasm of *Drosophila* germ cells

We selected *Drosophila* to study Top3b-TDRD3 in germ cells because this model has been extensively used to identify genes and pathways in gonads, with many findings applicable to mammals.^40,41^ We analyzed a previously described *Top3b*-knockout (KO) line^42^ (*Top3b*^*KO1*^) and two newly generated KO lines for *Top3b* or *Tdrd3* by CRISPR-Cas9 (Figures S1A–S1C). To study whether Top3b requires topoisomerase activity for its functions, we generated a knockin (KI) mutant, *Top3b*^*Y332F*^, by substituting the critical catalytic residue Tyr with phenylalanine, which inactivates the topoisomerase activity but retains RNA binding.^8,10^ Western blotting confirmed that *Y332F* expression is comparable to that of wild type (WT) (Figure S1B, right).

**Figure 1.**
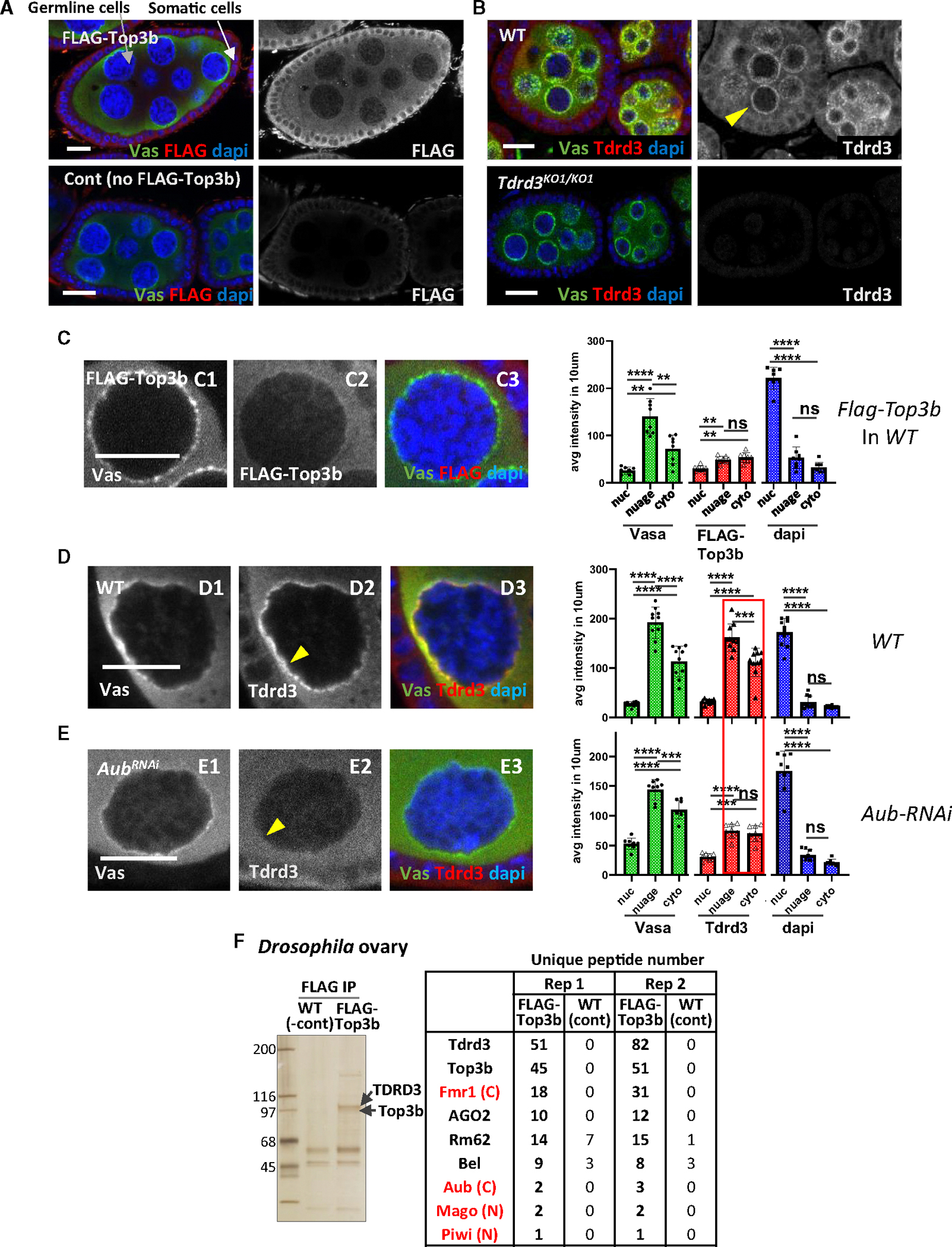
Top3b and TDRD3 are mainly localized in the cytoplasm in the ovary and interact with the piRNA machinery (A and B) Confocal images of Flag-Top3b (A) and TDRD3 (B) in *Drosophila* ovaries. The flies used are *Top3b*^*Flag*^ in the WT background (A, top), WT (*w*^*1118*^) (A, bottom, and B, top), and *Tdrd3* mutant (B, bottom). Stage 4–6 egg chambers were stained for Vasa (green), Flag-Top3b (red, A) or TDRD3 (red, B), and DAPI (blue). The arrows indicate germline nurse cells (gray arrow) and somatic follicle cells (white arrow). The yellow arrowhead indicates Vasa and Tdrd3 positive nuage. (C–E) High-magnification images of nurse cell-follicle boundary stained for Vasa, FLAG (C2), TDRD3 (D2 and E2), and DAPI. Arrowhead indicates Tdrd3 localization at nuage. Bar graphs quantify staining intensity in nucleus, nuage, and cytoplasm. (F) Silver staining of TDRD3 IP eluate from ovary lysates (left) and co-immunoprecipitated peptides with unique peptides detected by MS. All scale bars represent 10 μm. Data are represented as the mean ± SD. Statistical analysis was done by two-tailed t test (*p < 0.05, **p < 0.01, ***p < 0.001, and ****p < 0.0001).

Immunofluorescence in fly ovaries using FLAG-tagged Top3b (Flag-Top3b) showed that Top3b signals, detected by either FLAG (Figures 1A–1C) or its own antibody (Figure S2A), were higher in the cytoplasm than in the nucleus of both germline (nurse cells and oocytes; marked by Vasa) and somatic follicle cells (the outside layer of cells that surrounds the germ cells). Quantification confirmed that Flag-Top3b signals are significantly higher in the cytoplasm than in the nucleus (1.8-fold, *p* < 0.01) (Figure 1C, right). As controls, the co-staining with Vasa, known to localize in the cytoplasm, exhibits expected higher levels in the cytoplasm than in the nucleus (2.7-fold), whereas DAPI displays an opposite pattern—higher in the nucleus than in the cytoplasm (6.8-fold). Similar to Top3b, TDRD3 was 3.4-fold stronger in the cytoplasm than in the nucleus in germline cells (Figures 1B–1D). As negative controls, both Top3b and TDRD3 antibodies showed little signal in flies lacking these proteins (Figures 1A and 1B, bottom vs. top; Figure S2A, right vs. left). These data demonstrate that Top3b-TDRD3 primarily localizes in the cytoplasm, implying a possible role in RNA-related processes.

### TDRD3 is enriched in the nuage, where germline piRNA biogenesis occurs

We observed TDRD3 enrichment in the nuage, a perinuclear compartment where piRNA biogenesis (ping-pong cycle) occurs in germ cells (Figure 1B, arrowhead).^43^ High-magnification images confirmed strong TDRD3-Vasa co-localization in nuage (Figure 1D and D1–D3). Quantification further confirmed that TDRD3 signals were significantly higher in the nuage than in either the cytoplasm or the nucleus (*p* < 0.001) (Figure 1D, right). The enrichment of TDRD3 in the nuage resembles that of other Tudor-domain-containing proteins involved in piRNA biogenesis,^44^ implying that Top3b-TDRD3 may act similar to the other Tudor proteins in this process.

Unlike TDRD3, Top3b signals (with or without FLAG tag) are not enriched in the nuage vs. the cytoplasm (Figures 1C, C1–C3, and S2A). One possibility is that Top3b transiently interacts with nuage-localized TDRD3. Another is that the fraction of TDRD3 enriched in the nuage lacks Top3b.

### Localization of TDRD3 in the nuage depends on a piRNA biogenesis enzyme—Aub

We tested whether TDRD3 localization in the nuage depends on Aub, a key piRNA biogenesis enzyme that is mainly localized in the nuage and is essential for nuage localization of several piRNA pathway components, including a Tudor domain protein, Krimper.^45,46^ In *aub*-knockdown (KD) flies, TDRD3 enrichment in the nuage was abolished (Figures 1E and E1–E3), resembling Krimper, suggesting that TDRD3 may be recruited through a similar mechanism—via Tudor domain interactions with arginine-methylated Aub. In a reciprocal experiment, Aub localization was unaltered in *TDRD3*-KO flies (Figures S2B and S2C), consistent with a report that Aub localization in the nuage is guided by piRNA recognition of the RNA targets.^46^

We also examined whether Armitage (Armi) localization depends on Top3b or TDRD3, and vice versa, but obtained negative data (Figure S2D).

### Top3b-TDRD3 stably associates with Aub and other piRNA components in germ cells

We next used immunoprecipitation-coupled mass spectrometry (IP-MS) to identify proteins stably associating with Top3B-TDRD3 from *Drosophila* ovary lysates. FLAG IP from Flag-Top3b ovaries recovered two major polypeptides with molecular weights similar to those of Top3b and TDRD3 (Figure 1F, left), which were absent in control flies lacking Flag-Top3b (Figure 1F, right).^10,16^ Two independent replicates of IP-MS confirmed these proteins to be Top3b and TDRD3, with 45 and 82 unique peptides for each protein, whereas mock IP-MS from the control flies yielded none, indicating that our IP specifically isolated the Top3b-TDRD3 complex (Figure 1F, right table).

To filter specific interactors with Top3b-TDRD3 from those that cross-react with the antibody beads, we selected proteins with >2-fold unique peptide enrichment in Flag-Top3b vs. those of control flies in both replicates. Importantly, Aub and Piwi, which belong to the same PIWI Argonaute family and are major components of piRNA pathways,^47^ met this cutoff, suggesting that Top3b-TDRD3 may act in piRNA processes. Gene Ontology (GO)-term analysis supported this, linking Flag-Top3b-associated proteins to RNA interference and PTGS, which includes the piRNA pathway (Figure S2E).

**Figure 2.**
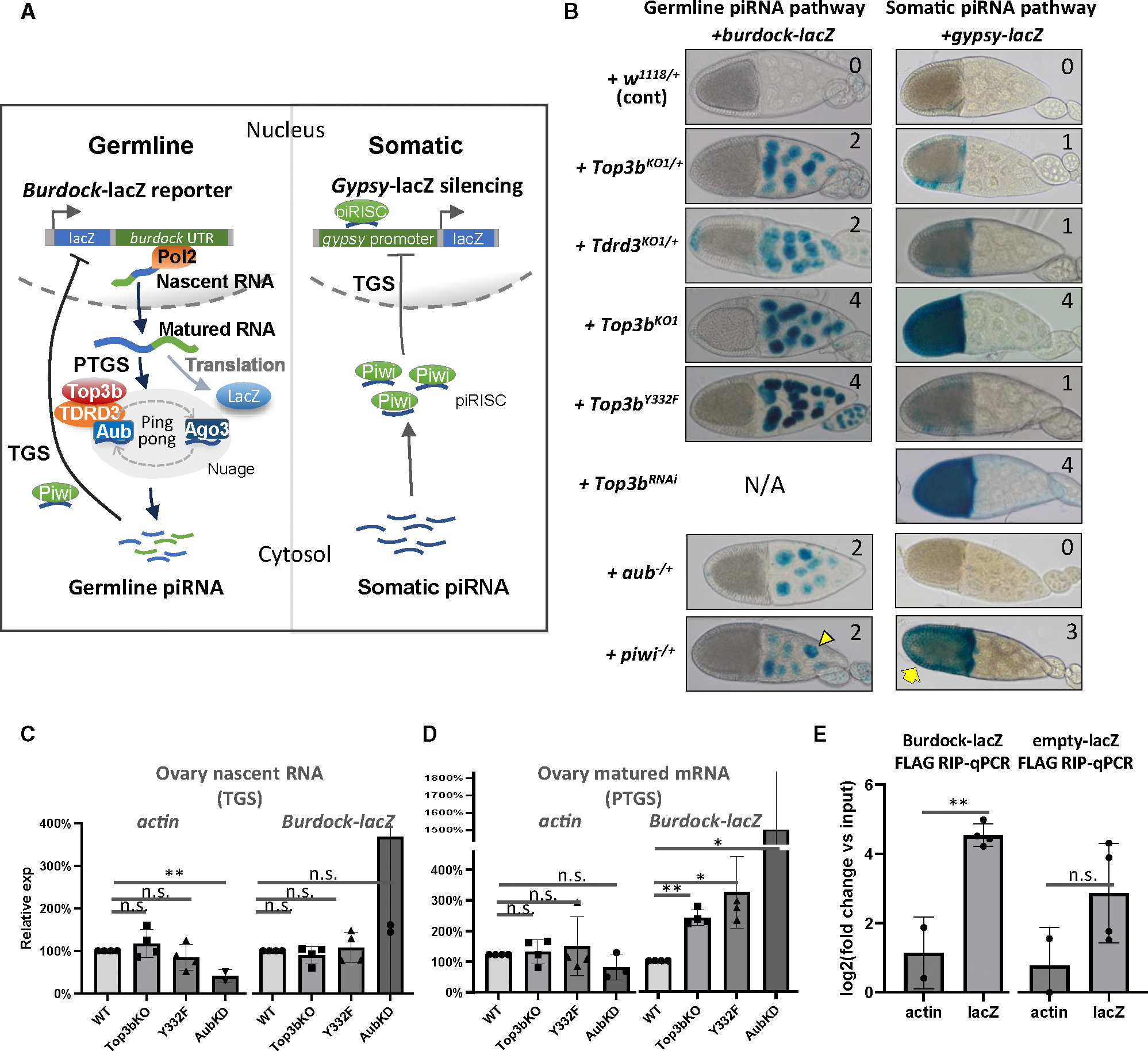
The Top3b-TDRD3 complex participates in both somatic and germline piRNA pathways to silence transposons (A) Schematic of *burdock*- and *gypsy-lacZ* reporter silencing assay. (B) Representative β-gal staining of *burdock-lacZ* and *gypsy-lacZ* reporters in *Top3b*, *Tdrd3*, *aub*, and *piwi* mutants. Genotypes are listed on the left. β-gal staining intensity is quantitated from 0 to 4 on the right. (C–E) RT-qPCR for *actin* and *burdock-lacZ* expression. Nascent RNAs (C) and matured mRNAs (D) were extracted for the assay. (E) RT-qPCR quantification for *actin* and *lacZ* RNAs from FLAG RIP. Data are represented as the mean ± SD. Statistical analysis was done by two-tailed t test (*p < 0.05 and **p < 0.01).

Aub acts in the germline cytoplasm to promote ping-pong piRNA amplification, which simultaneously cleaves TE transcripts, resulting in PTGS of TEs^47,48^ (Figure 2A). Conversely, Piwi functions in both the cytoplasm, as a part of the primary piRNA processing complex, and the nucleus, as a component of the piRISC (piRNA-induced silencing complex) that mediates TGS of TEs in *trans.*^47,49,50^ Our findings that Top3b-TDRD3 predominantly localizes in the cytoplasm suggest that the complex mainly works with Aub in piRNA biogenesis and PTGS.

### Top3b-TDRD3 stably associates with primary piRNA processing enzymes

Two different piRNA pathways have been identified in *Drosophila* germline and somatic follicle cells: the ping-pong pathway is present only in the former, whereas primary piRNA processing occurs in both^51^ (see Figure 2A). To determine whether Top3b-TDRD3 interacts with primary pathway components, we performed reciprocal TDRD3 and Piwi IP-MS and IP-western using lysates from the ovarian somatic sheath cell line OSS.^52^ Top3b-TDRD3 co-immunoprecipitated with the cytoplasmic primary piRNA processing complex containing Piwi-Armi-YB-SpnE (Figures S2F and S2G), as well as nuclear piRNA components (Panx, Nxf1/2, and HP1/2) (Figure S2F). These data suggest that Top3b-TDRD3 may promote both piRNA processes in the cytoplasm and TGS of TEs in the nucleus in somatic ovarian cells.

We used the same IP-MS approach in mouse testis lysates and found that Top3b-TDRD3 associates with Aub/Piwi orthologs and other piRNA components (Figure S2H), suggesting that the association between Top3b-TDRD3 and the piRNA machinery is conserved.

### Top3b-TDRD3 facilitates both ping-pong and somatic silencing of TEs

The findings that Top3b-TDRD3 associates with both ping-pong and primary piRNA pathway components prompted us to use two pathway-specific reporters to investigate whether the complex can silence TEs in each pathway. The ping-pong pathway reporter *burdock*-lacZ^51,53^ contains a 3ʹ UTR fragment of a germline transposon, *burdock*, fused to the 3ʹ UTR of lacZ (Figure 2A, left). Because lacZ expression is driven by the *nanos* promoter, this reporter is sensitive to both PTGS and TGS mediated by piRNAs targeting the 3ʹ region of *burdock.*^53^ In contrast, the primary pathway reporter *gypsy*-lacZ^54^ contains the somatic *gypsy* promoter fused 5ʹ of the lacZ gene, making it responsive to TGS mediated by piRNAs targeting the *gypsy* promoter (Figure 2A, right). Because this reporter detects the endpoint of the piRNA pathway, it can also capture defects in earlier steps, including piRNA biogenesis.

As a negative control, flies carrying either reporter in the WT background exhibited no LacZ staining (Figure 2B, top, blue scale 0; see scales in Figure S3A), indicating that both piRNA pathways function normally. As positive controls, flies carrying *burdock*-lacZ in the *aub*^+*/*−^ mutant or *gypsy*-lacZ in the *piwi*^+*/*−^ mutant background exhibited strong LacZ straining in germline nurse cells or somatic follicle cells, respectively (Figure 2B, bottom left, scale 2; right, scale 3), consistent with findings that mutations in these genes disrupt TE silencing.^53^ Notably, in *Top3b* homozygous mutant or *Top3b* KD flies, both reporters were maximally activated in germline or somatic ovarian cells, respectively (Figure 2B, scale 4), indicating that Top3b is critical for both pathways.

Moreover, in the homozygous *Top3b*^*Y332F*^ catalytic mutant, the *burdock* reporter was maximally activated (scale 4), identical to the *Top3b*^*KO1*^ mutant, suggesting that topoisomerase activity is required for Top3b to promote PTGS and/or TGS of the reporter. In contrast, the *gypsy* reporter was only mildly activated (scale 1)—higher than WT but lower than *Top3b*^*KO1*^, indicating that topoisomerase activity contributes to, but is not absolutely essential for, TGS by the primary piRNA pathway.

To exclude the possibility that the *burdock*-LacZ silencing was due to lacZ rather than the *burdock* sequence, we examined an empty-lacZ reporter lacking *burdock* (Figures S3B–S3D). This reporter shows strong LacZ straining in WT flies (scores 3–4), indicating that the silencing requires *burdock*. *Top3b* or *TDRD3* mutants exhibited lacZ expression comparable to that of WT flies (Figures S3C and S3D), ruling out the lacZ sequence as the silencing target of Top3b-TDRD3.

### Top3b and its topoisomerase activity facilitate PTGS of the *burdock* transposon reporter

To determine whether Top3b acts at TGS or PTGS of the *burdock*-lacZ reporter, we analyzed nascent and mature transcripts in the *Top3b* mutant. If Top3b acts at TGS, the nascent *burdock*-lacZ transcript level should increase in the *Top3b* mutant (Figure 2A), and if Top3b acts at PTGS, the nascent transcript level should remain unchanged, while the mature transcript should increase. Our analyses showed no significant changes in nascent (*p* > 0.05), but 2.5- to 3-fold increase in mature, transcripts (*p* < 0.05) (Figures 2C and 2D) in *Top3b*^*KO1*^- and *Top3b*^*Y332F*^-KI flies. As a positive control, flies depleted of Aub, which promotes PTGS, exhibited the same pattern. As a negative control, actin transcripts remained unchanged across all mutants (Figures 2C and 2D). These data suggest that Top3b and its topoisomerase activity promote PTGS but not TGS of the *burdock*-lacZ reporter.

For *gypsy*-lacZ, both nascent and mature transcripts increased 4- to 24-fold in *Top3b*-KO flies (Figure S3E), suggesting that Top3b promotes TGS of this somatic TE reporter.

### Top3b binds the transposon reporter RNA

To determine whether Top3b binds *burdock*-lacZ mRNA, we performed RNA-immunoprecipitation (RIP)-coupled qPCR using ovary lysates from *Flag-Top3b/Top3b*^*KO1*^ heterozygous mutant flies carrying the *burdock*-lacZ reporter (Figure 2E). Flag-Top3b RIP showed 5-fold enrichment of *burdock*-lacZ mRNA over *actin* mRNA (*p* < 0.01, 5-fold) (Figure 2E), suggesting selective binding to the reporter RNA. Moreover, the empty-lacZ reporter, which lacks the *burdock* sequence, showed no significant enrichment (Figure 2E), suggesting that Top3b likely binds the reporter transcript via the *burdock* sequence.

Genetic interactions with piRNA processing enzymes further support Top3b’s role in TE silencing (Figures S3F–S3K). Specifically, *Top3b* positively interacts with *piwi*, *zuc*, and a somatic piRNA locus to silence the *gypsy*-lacZ reporter (Figures S3I and S3J), suggesting that Top3b may work with piRNA and its processing enzymes to silence TEs.

### Inactivation of Top3b de-silences endogenous TEs

**Figure 3.**
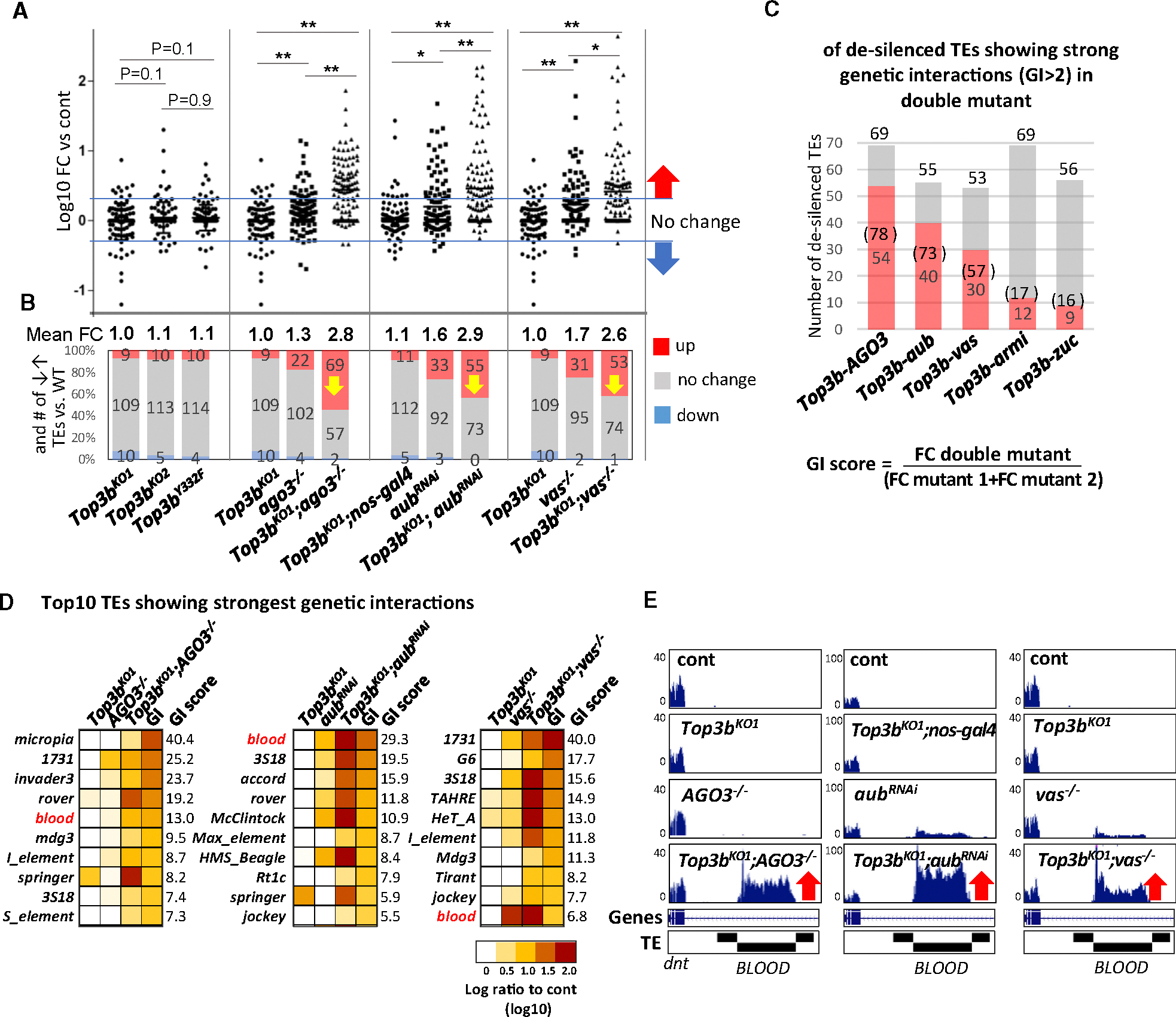
*Top3b* and piRNA components genetically interact to silence endogenous TEs (A) Whisker plots showing up- and downregulated transposons in three alleles of *Top3b* mutants and key piRNA component mutants from RNA-seq. Mean fold change of overall TEs is shown at the bottom. Data are represented as the mean ± SD. Statistical analysis was done by two-tailed t test (*p < 0.05 and **p < 0.01). (B) Graphs showing the numbers of TEs that are decreased (blue), unchanged (gray), or increased (red) (cutoff: 2-fold). Numbers represent TE count. (C) Number and percentage of de-silenced TEs in five genetic interaction assays. Red represents GI > 2. GI score is calculated as fold change (double mutant vs. cont)/fold change (sum of single mutant vs. cont). (D) Heatmap of RNA-seq showing top 10 strong genetic interactions between *Top3b* and piRNA components. See also Table S2. (E) Genome browser view of *blood* transposon by RNA-seq, highlighting robust increase in *blood* RNA levels in all double mutants (red arrows).

We investigated whether Top3b inactivation can de-silence endogenous TEs, using RNA sequencing (RNA-seq) to identify TE-derived transcripts elevated in *Top3b* mutant vs. WT fly ovaries by at least 2-fold. As positive controls, we analyzed five mutants of the piRNA pathway (*ago3*, *aub*, *vasa*, *armi*, and *zuc*) and observed higher mean levels of TE transcripts (fold change [FC]: 1.3–3.1) and larger numbers of de-silenced TEs (22–62) than extra-silenced TEs (those showing lower expression than WT) (0–4) (Figures 3A, 3B, and S4A; Table S1).

RNA-seq analysis of two *Top3b*-KO and one *Top3b-Y332F*-KI flies showed mean TE transcript levels with smaller FCs (1.0–1.1) than those in piRNA mutants (Figure 3A), suggesting that Top3b plays a lesser role than piRNA components in TE silencing. The numbers of de-silenced TEs in *Top3b*^*KO1*^ mutants under the *nos-GAL4* background, or in *Top3b*^*KO2*^ and *Top3b-Y332F* mutants (9–11), were about twice those of extra-silenced TEs (4–5). In contrast, the numbers of de-silenced and extra-silenced TEs were comparable (9 vs. 10) in *Top3b*^*KO1*^ mutants under the *w*^*1118*^ background (10) (Figure 3B). RT-qPCR confirmed increased expression of *gypsy6* and *tahre* in *Top3b*^*KO1*^ (Figure S4B). Thus, all three assays (reporter, RNA-seq, and RT-qPCR) show that Top3b is required for silencing a subset of TEs.

### *Top3b* interacts with piRNA components to silence endogenous TEs

We examined whether *Top3b* genetically interacts with piRNA components to silence endogenous TEs by comparing mean TE levels in single vs. double mutants. For three piRNA components (*ago3*, *aub*, and *vasa*), their double mutants with *Top3b*^*KO1*^ showed significantly higher TE expression (relative to *w*^*1118*^ control, 60%–120%; *p* < 0.05) compared with their respective single mutants (Figure 3A; Table S1). Moreover, the numbers of desilenced TEs in these double mutants were more than the sum of their single mutants (Figure 3B; Table S1), suggesting a strong genetic interaction with these three piRNA components.

For the other two piRNA components (*armi* and *zuc*), no obvious genetic interactions with *Top3b* were detected (Figure S4A), likely because de-silencing of TEs in these two single mutants was already too strong.

### *Top3b* interacts with piRNA components to silence a subset of TE families

To quantify genetic interactions at the individual TE level, we established a genetic interaction score (GI score) for each TE by calculating the ratio between the FCs of the double mutants vs. the sum of FCs of their single mutants. If this ratio is larger than 1, the de-silencing of this TE in the double mutant is stronger than the combined actions of each single mutant—an indication of positive genetic interactions. We used GI scores >2 to identify TEs regulated by strong positive genetic interactions. In the analysis of *Top3b* interactions with *ago3*, *aub*, or *vasa*, the majority of de-silenced TEs (57%–78%) showed strong genetic interactions (Figure 3C). In contrast, *armi* and *zuc* exhibited weak or no interactions with *Top3b* (16%–17%, Figure S4C).

Heatmaps confirmed much higher TE expression in double vs. single mutants (Figure 3D and S4D), with GI scores as high as 40. A fraction of these TEs, such as *blood*, showed interactions across multiple double mutants (Figures 3E and S4E). RT-qPCR analysis confirmed that several representative TEs display higher expression in double mutants than in their signal mutants (Figure S4B). The data support the conclusion that *Top3b* and piRNA components cooperate to silence endogenous TEs.

### Top3b preferentially promotes silencing of long and highly expressed TEs

**Figure 4.**
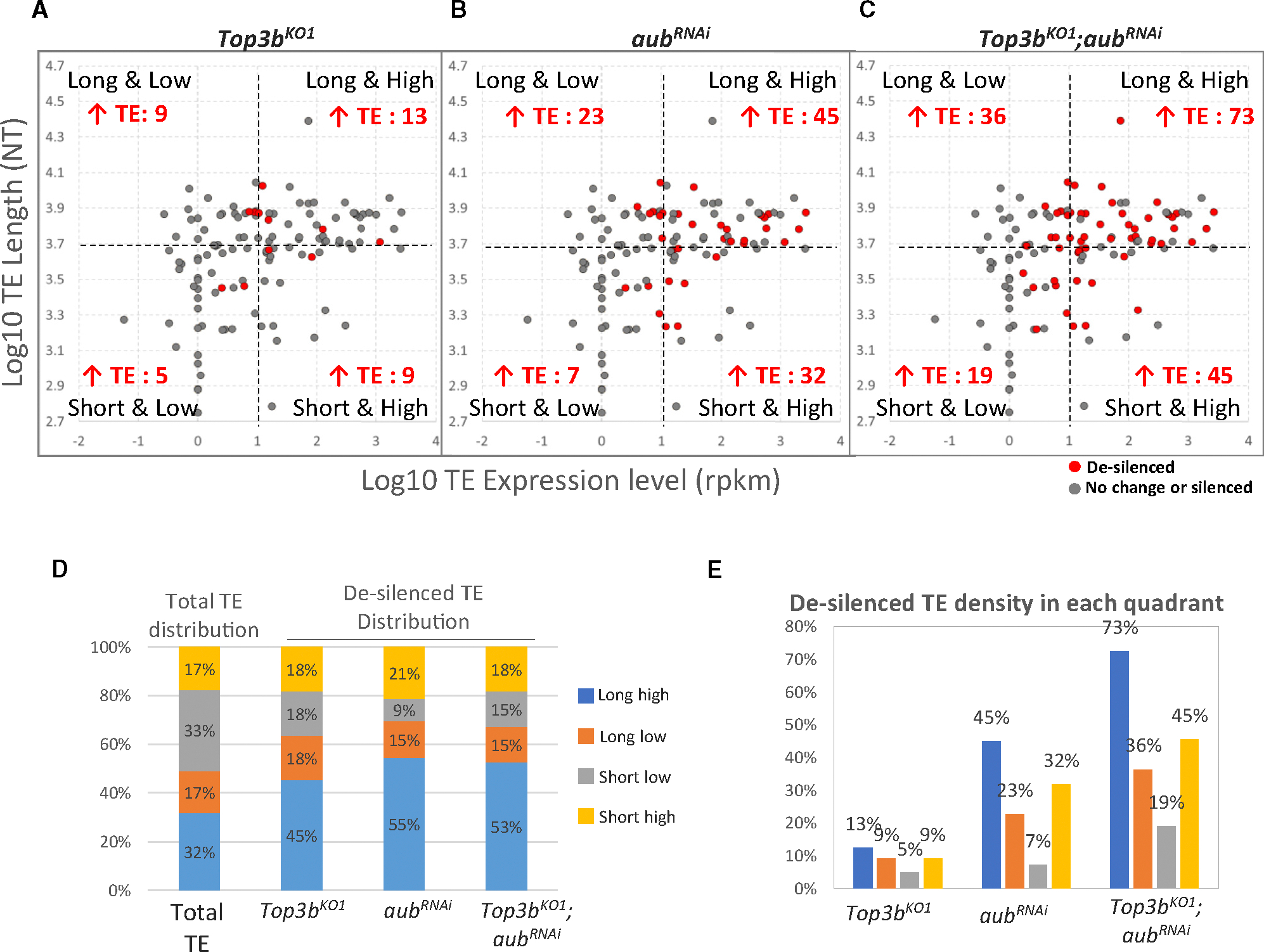
Top3b preferentially promotes silencing of long and highly expressed TEs (A–D) Scatterplots (A–C) showing distribution of de-silenced or total TEs from single and double mutants of *Top3b* and *aub*. TE profiles are divided into four quadrants by length (log10 length, y axis) and expression level (log10 rpkm, x axis): long and high, long and low, short and low, and short and high. Cutoffs are average log length of 3.7 (~5,000 NTs) and log expression level of 1 (10 rpkm). Red marks indicate de-silenced TEs within each quadrant, summarized in (D). (D) Distribution of de-silenced TEs based on the criteria in (A–C). (E) Density of de-silenced TEs in each quadrant.

Topoisomerases, including Top3b, preferentially enhance the transcription of long and highly transcribed genes,^13,14,55–57^ which are believed to produce more DNA topological problems requiring topoisomerases to solve.^55^ We hypothesized that long and highly expressed transposon RNAs may similarly produce more topological problems requiring Top3b. To test this, we divided de-silenced TEs from *Top3b* and *aub* mutants into four quadrants by their length (long or short) and expression level (high or low) and compared them with total TEs (Figures 4A–4C). We found that the distribution of de-silenced TEs in the long-high quadrant for the single and double mutants was 1.4- to 1.7-fold higher (45%, 55%, and 53%), whereas that in the short-low quadrant was 1.8–3.7-fold lower (18%, 9%, and 15%), than that of total TEs in these quadrants (32% and 33%, respectively) (Figure 4D). In contrast, distribution in the short-high and long-low quadrants was similar to that of total TEs. Moreover, the density of de-silenced of TEs, defined as the percentage of desilenced TEs among total TEs within a quadrant, was highest in the long-high, but lowest in short-low for all mutants (Figures 4A–4C and 4E), with a 2.6- to 6.4-fold difference. These data suggest that Top3b cooperates with Aub to preferentially silence long and highly expressed TEs.

Analysis of *Top3b*-*ago3* or *Top3b-vasa* mutants yielded similar results (Figures S4F–S4I). Notably, the de-silenced TE density in double mutants was always greater than the sum of the single mutants across all quadrants (Figures 4D, S4F, and S4H), suggesting that *Top3b* genetically interacts with multiple piRNA processing enzymes to silence TEs regardless of their length and expression level.

### *Top3b* interacts with *aub* to promote ping-pong piRNA biogenesis in germ cells

**Figure 5.**
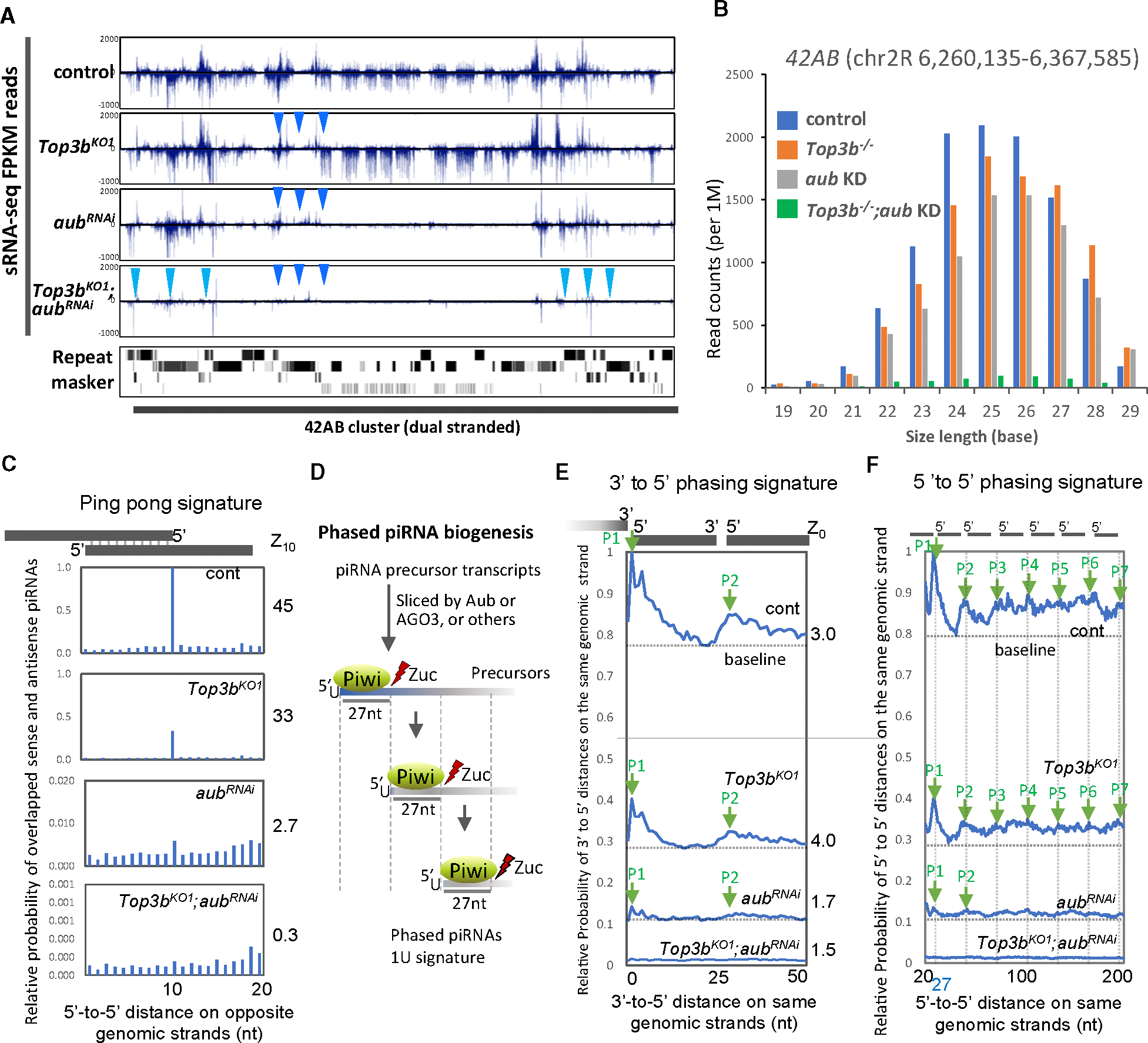
*Top3b* works with *aub* to promote both ping-pong and primary piRNA biogenesis pathways (A) A genome browser view of piRNA reads from sRNA-seq of mutants or WT flies (top four rows) on the *42AB* piRNA cluster. Bottom row shows RepeatMasker reference for TE identification. (B) Bar graph representing sRNA reads distribution (19–29 nt) within the *42AB* locus. (C) Graphs showing ping-pong signatures (5ʹ−5ʹ sense-antisense distance) of *42AB* locus-mapped piRNAs, calculated by PPmeter. Ping-pong scores (Z_10_) are listed on the right. (D) Cartoon illustrating how piRNAs with phasing and 1U signatures are produced by the primary biogenesis pathway. (E and F) Graphs showing 3ʹ-to-5ʹ and 5ʹ-to-5ʹ phasing signatures, respectively, for piRNAs mapped to the *42AB* locus in different mutants or WT flies. Phasing scores (Z_0_) are the average *Z* score (*p* < 0.05) for the 3ʹ-to-5ʹ distance. Peaks with 27-bp intervals are marked by green arrows. The baseline of each profile is the average value at the valley between peak 1 and peak 2, used to measure peak height.

The five piRNA components genetically interacting with *Top3b* in TE silencing are essential for piRNA biogenesis in germ cells, suggesting that Top3b likely works with them in the same process. To test this, we performed small RNA-seq (sRNA-seq) on ovaries from *Top3b* and *aub* single and double mutants, as the products of these genes biochemically and genetically interact (Figures 1F and 3). In WT, the sRNA reads predominantly mapped to germline and somatic piRNA clusters, with sizes peaking in the same range (23–28 nt) as those of piRNAs^58,59^ (Figures 5A, 5B, S5A, and S5B). Most reads (>70%) matched piRNAs bound by Piwi, Aub, or Ago3 or their different combinations^60^ (Figure S5D). Furthermore, these sRNA reads exhibit four piRNA signatures (see below), indicating they are primarily piRNAs.

First, sRNA reads from WT flies exhibit a ping-pong signature with a peak of 10 nt overlap between sense and antisense strands (Figure 5C), suggesting a large fraction of these reads are piRNAs from the ping-pong cycle. In *aub*-KD flies, this signature is strongly disrupted, evidenced by a 100-fold reduction in this peak (1–0.01) and a 15-fold decrease in the ping-pong score (Figure 5C, *Z*_10_ = 45 vs. 2.7 in WT vs. *aub*-KD flies). In addition, the levels of sRNA reads are reduced by 5%–50% at the germline piRNA loci *42AB* and *80F* (Figures 5A, 5B, and S5C), but not at the somatic piRNA cluster *flam* (Figures S5A and S5B). All these results are in agreement with reports that Aub is critical for germline but not somatic piRNA biogenesis.^51^

The *Top3b* mutant shows a reduction in sRNA reads at several loci within the germline piRNA cluster (Figure 5A), reductions at several but not every piRNA length (Figure 5B, right), and reduction in the ping-pong signature, reflected by a decrease of the 10-nt peak (1–0.4) and ping-pong score (Figure 5C, *Z*_10_ = 45 vs. 33 in WT vs. mutant). In contrast, the mutant shows no reduction in sRNA reads at the *flam* cluster (Figure S5A). These features mimic those of *aub*-KD flies, suggesting that Top3b resembles Aub in promoting ping-pong piRNA biogenesis, although its effects are smaller than those of the latter.

Importantly, the *Top3b-aub* double mutant shows stronger reduction in piRNA reads than their single mutants at the germline *42AB* or *80F* cluster (Figures 5A and 5B, left; Figure S5C), but not the *flam* piRNA cluster (Figures S5A and S5B). Moreover, the mutant exhibits a near total loss of ping-pong signature in its piRNAs, evidenced by the diminished 10-nt peak and 150-fold decrease in the ping-pong score (Figure 5C, *Z*_10_ = 45 vs. 0.3 in WT vs. double mutant). These data indicate that *Top3b* genetically interacts with *aub* to promote ping-pong piRNA biogenesis and TE silencing in germ cells.

We then analyzed the 10A signature,^47^ another hallmark of piRNAs produced by the ping-pong cycle, but found that this signature was statistically unaltered in total piRNAs between *Top3b-aub* mutants and WT (Figure S5F). We reasoned that the effect of *Top3b-aub* mutations on this signature may be masked by the more abundant primary piRNAs that lack the 10A bias. To circumvent this problem, we calculated the 10A% for Ago3-bound piRNAs (Figure S5E), which are enriched for sense piRNAs with 10A signature.^60,61^ Consistent with earlier reports, we observed that 10A% was increased from about 25% to 65% in Ago3-bound piRNAs vs. total piRNAs for WT flies (Figure S5F vs. S5G). Notably, 10A% of Ago3-bound piRNAs shows a small but significant reduction (7%–9%; *p* < 0.05) in the double vs. either single mutant or WT on the sense strand, but statistically insignificant reductions on the antisense or both strands (Figures S5G and S5H), suggesting a modest disruption of this signature on sense piRNAs in the double mutant. The data provide additional evidence that Top3b interacts with Aub to promote ping-pong piRNA biogenesis.

### Top3b inactivation disrupts signatures of the primary piRNA pathway in the absence of Aub

Next, our analysis revealed that total piRNAs or those mapped to the *42AB* locus from WT flies display typical 3ʹ-to-5ʹ and 5ʹ-to-5ʹ phasing signatures of piRNAs from the primary biogenesis pathway (Figures 5D–5F and S5I), with an average of 27-bp periodicity when these reads are mapped to the same genomic strand,^60,62–64^ indicating that a large fraction of the reads is produced from this pathway. In *aub*-KD flies, these phasing signatures are reduced, as evidenced by drastically decreased peak heights (>10-fold), fewer peaks in the 5ʹ-to-5ʹ graph, and smaller phasing scores (Z_0_ = 1.7 vs. 3.0 in mutant vs. WT) (Figures 5D and 5E). These findings support Aub’s role in piRNA pathway initiation and progressive steps in germ cells.^65^ In *Top3b*-KO flies, peak heights are reduced by 2-fold in both graphs, but phasing scores remain unchanged (Z_0_ = 4.0 vs. 3.0 in mutant vs. WT), suggesting that the piRNAs produced have normal phasing but at reduced levels. Importantly, in the *Top3b-aub* double mutant, the phasing signatures are more severely disrupted, with no detectable phased piRNA peaks and a 2-fold decrease in phasing score (Z_0_ = 1.5 vs. 3.0 in mutant vs. WT) (Figure 5E). Moreover, we observed a stronger distance-dependent decline of signals in the 5ʹ-to-5ʹ graph for total piRNAs from the *Top3b-aub* double compared with their single mutants (Figure S5I). These findings suggest that Top3b plays a minor role in initiating phased piRNA biogenesis when Aub is present. However, in the absence of Aub, Top3b plays a larger role, likely by activating alternative mechanisms independent of Aub.

Our further analysis showed that sRNA reads from WT and different mutant flies exhibit the 1U signature, another hallmark of primary piRNA biogenesis^47,48^ (Figure 5D), as 78%–80% of TE-mapped reads have uridine at their 5ʹ position (Figure S5J, left). In addition, the 1U% in either mutant or WT flies is increased by about 2% in those mapped to the antisense strand of TEs (Figure S5J, right), but decreased by about 5%–12% in those mapped to the sense strand (Figure S5J, middle), in accord with reports that 1U is enriched in antisense piRNAs.^47^ We then calculated the differences in 1U% between different mutants and WT on sense, antisense, or both strands of piRNAs (Figures S5J and S5K). For *Top3b* and *aub* single mutants, their 1U% is not significantly different from that of WT for all strands of piRNAs. Notably, for the *Top3b-aub* double mutant, the 1U% of sense piRNAs shows 7.3% reduction vs. WT, which reaches statistical significance (*p* < 0.05), whereas those of antisense or both strands display 3.5-fold smaller reduction (2.3% each), which is below the significance cutoff (*p* = 0.08 and 0.06) (Figures S5J and S5K). Moreover, the 1U% of the sense piRNAs also displays stronger reduction than that of the antisense when comparing the double mutant vs. each single mutant. These results suggest that Top3b cooperates with Aub to preferentially enhance the primary biogenesis of sense piRNAs. Furthermore, the 1U signatures of both strands are significantly lower in the double mutant than in the *aub* single mutant (Figures S5J and S5K), supporting the idea that Top3b activates alternative mechanisms to promote the primary pathway in the absence of Aub.

The findings that *Top3b-aub* double mutants display stronger disruption of four different piRNA signatures than their single mutants imply that the former should also show stronger reduction in piRNA levels. Consistent with this prediction, the levels of piRNAs mapped to both strands of consensus TEs showed stronger reduction in the double than in each single mutant (Figures S6A and S6B; Table S3). Moreover, the numbers of reduced piRNAs are also more in the former than in the latter (Figures S6C and S6D). Together, these data indicate that Top3b works with Aub to promote piRNA biogenesis.

### *Top3b* preferentially enhances biogenesis of sense piRNAs

We investigated whether Top3b binds piRNAs like PIWI-clade proteins, but detected no binding (Figure S6F). Instead, Top3b binds high molecular weight mRNAs, which was confirmed by RIP sequencing (RIP-seq) (data not shown), consistent with our earlier findings in human cell lines.^10,11^ Our RIP-seq also revealed that Top3b binds several RNAs derived from TEs desilenced in *Top3b* single or double mutants (Figures S6G and S6H), suggesting that Top3b may interact with TE-derived RNAs and promote their silencing. Because TE-derived RNAs are precursors of sense piRNAs, which are cleaved by Aub to initiate ping-pong and primary biogenesis pathways^47^ (see Figure 7I for cartoon), our data support a hypothesis that Top3b may work with Aub to process TE transcripts into sense piRNAs (Figure 7I).

**Figure 6.**
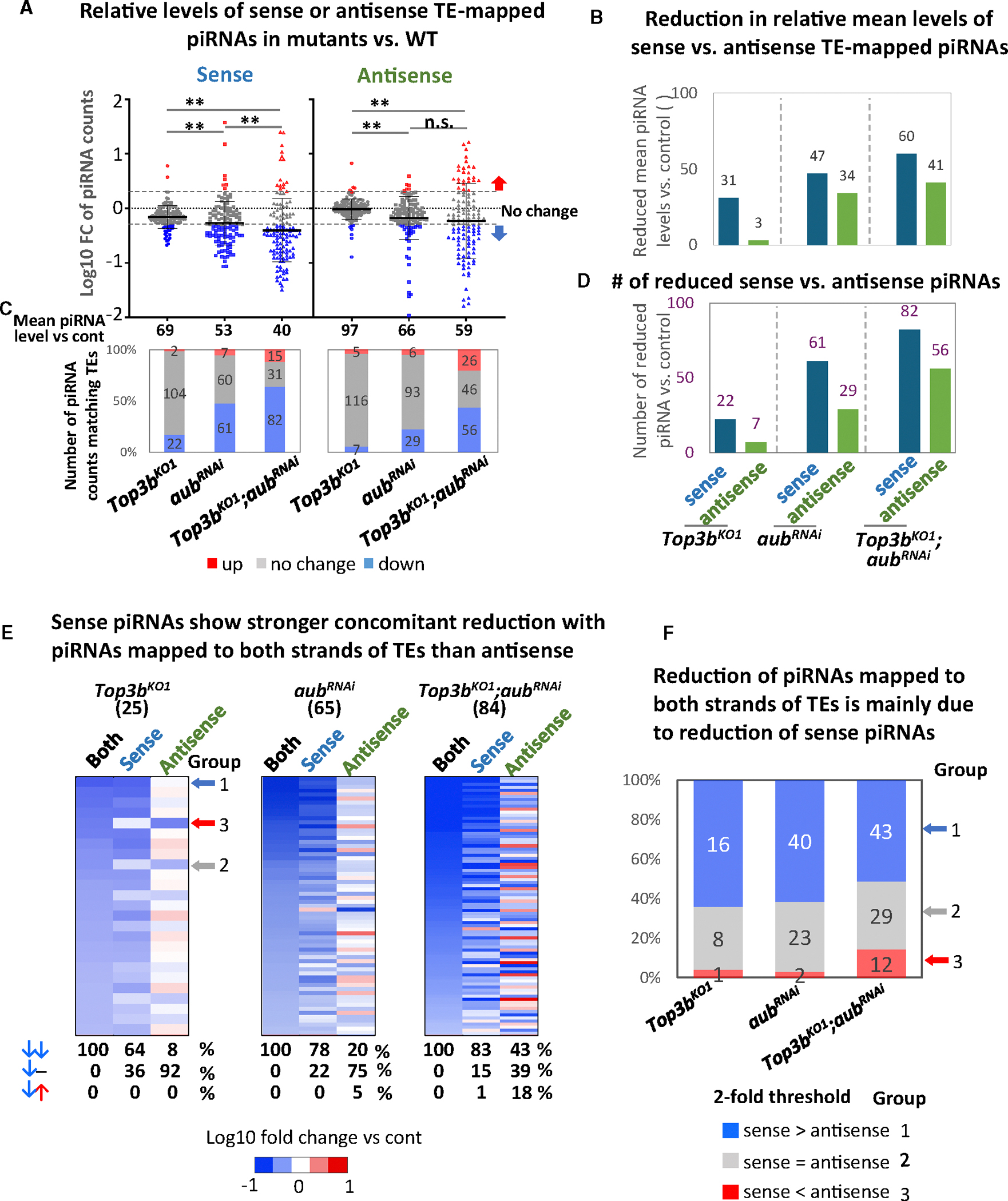
Top3b works with Aub to preferentially enhance biogenesis of sense piRNAs (A) Whisker plots showing log10 fold changes of TE-mapped piRNA counts in *Top3b* and *aub*^*RNAi*^ single or double mutants, separated by sense and antisense. Each data point represents a group of piRNAs mapped to one TE. Up-/downregulated TE-mapped piRNAs (2-fold cutoff) are marked by red or blue arrows. Mean relative piRNA levels are listed below, with WT set to 100%. Data are represented as the mean ± SD. Statistical analysis was done by two-tailed t test (**p < 0.01). (B) Graph showing reduction in relative mean levels of sense vs. antisense TE-mapped piRNAs. (C) A graph showing the number of upregulated, unchanged, or downregulated piRNA-mapped TEs (sense, antisense, or both strands) in single and double mutants vs. WT flies. (D) Graph showing reduced numbers of TE-mapped piRNAs separated by sense, antisense, or both strands. (E) Heatmaps showing the concomitant reduction in sense or antisense piRNAs vs. total piRNAs of both strands in single or double mutants. The numbers of reduced piRNAs are indicated on top. Scale indicator represents log10 fold change of mutant vs. control. The table below the heatmaps summarizes the percentages of TE-mapped piRNAs showing same or opposite directions of alterations (marked by arrows) or no concomitant alteration (marked with “−”). The three arrows on the right of the first graph represent three examples of groups 1–3, respectively. (F) Graph showing relative abundance of sense or antisense piRNAs in TE-mapped piRNAs reduced in single or double mutants. A 2-fold cutoff was used to distinguish the three groups of piRNAs as indicated.

To examine this hypothesis, we separated TE-mapped piRNAs into sense and antisense and analyzed each subset as we did for TEs (Figures 6A and 6B). Each data point represents piRNAs aligned to either the sense or the antisense strand of one transposon. The results show that the *Top3b* mutant displays stronger reduction of sense than of antisense piRNAs. First, the mean level of sense piRNAs (69%) is ~30% lower than in control flies (set as 100%), while antisense (97%) shows little reduction (Figure 6A). Second, sense piRNA levels in *Top3b*-KO vs. WT are reduced 10-fold more than antisense piRNAs (31% vs. 3%) (Figure 6B). Third, three times more sense piRNAs are reduced compared to antisense (22 vs. 7) (Figures 6C and 6D). Fourth, the reduction in piRNAs mapped to both strands of TEs in the *Top3b* mutant (referred to as “both strands”) is mainly due to sense piRNAs, as 8-fold more sense than antisense piRNAs show concomitant reduction with those of both strands (64% vs. 8%) (Figure 6E, left). Moreover, we divided the 25 piRNAs of both strands reduced in the *Top3b* mutant into three groups (1–3), with each group consisting of sense piRNAs that have stronger, comparable, or weaker reduction than antisense piRNAs, respectively (2-fold cutoff) (Figures 6E and 6F). Most of the reduced piRNAs belong to group 1 (60%), whereas only 2.5% belong to group 3, suggesting that the reduction of sense but not antisense piRNA accounts for the reduction of most piRNAs in *Top3b* mutant.

### *Top3b* and *aub* genetically interact to preferentially enhance sense piRNA biogenesis

Our analysis of sense and antisense piRNAs in *aub* single or *Top3b-aub* double mutants vs. WT revealed stronger reduction of sense than of antisense piRNAs (Figures 6A–6F), similar to the *Top3b* mutant, consistent with Aub’s role in initiating piRNA biogenesis from TE transcripts.^60,63^

We then compared the sense and antisense piRNAs between *Top3b-aub* double and each single mutant and observed both similarities and differences between the two strands (Figures 6A–6D). The similarities include that the mean levels of either sense or antisense piRNAs of the double mutant are all lower than those of each single mutant (Figures 6A and 6B) and that the numbers of reduced sense or antisense piRNAs in the double mutant are also larger than those of single mutants (Figures 6C and 6D). These data suggest that *Top3b* interacts with *aub* to enhance biogenesis of both sense and antisense piRNAs. The differences include that the mean piRNA levels between the double and the *aub* single mutants significantly differ only for sense and not antisense piRNAs (Figure 6A). Moreover, sense piRNAs displayed lower mean levels (Figure 6A), bigger reduction in mean levels (Figure 6B), and larger numbers of reduced piRNAs (Figure 6B–6D) than antisense in the double mutant. These data further support that Top3b and Aub preferentially enhance sense piRNAs biogenesis.

We investigated whether de-silenced TEs in *Top3b-aub* mutants are due to reduced biogenesis of their matching piRNAs and obtained evidence supporting a causal relationship (Figures S6I–S6M; Tables S4–S7).

### *Top3b* and *Tdrd3* mutant *Drosophila* have defective fertility and oogenesis

**Figure 7.**
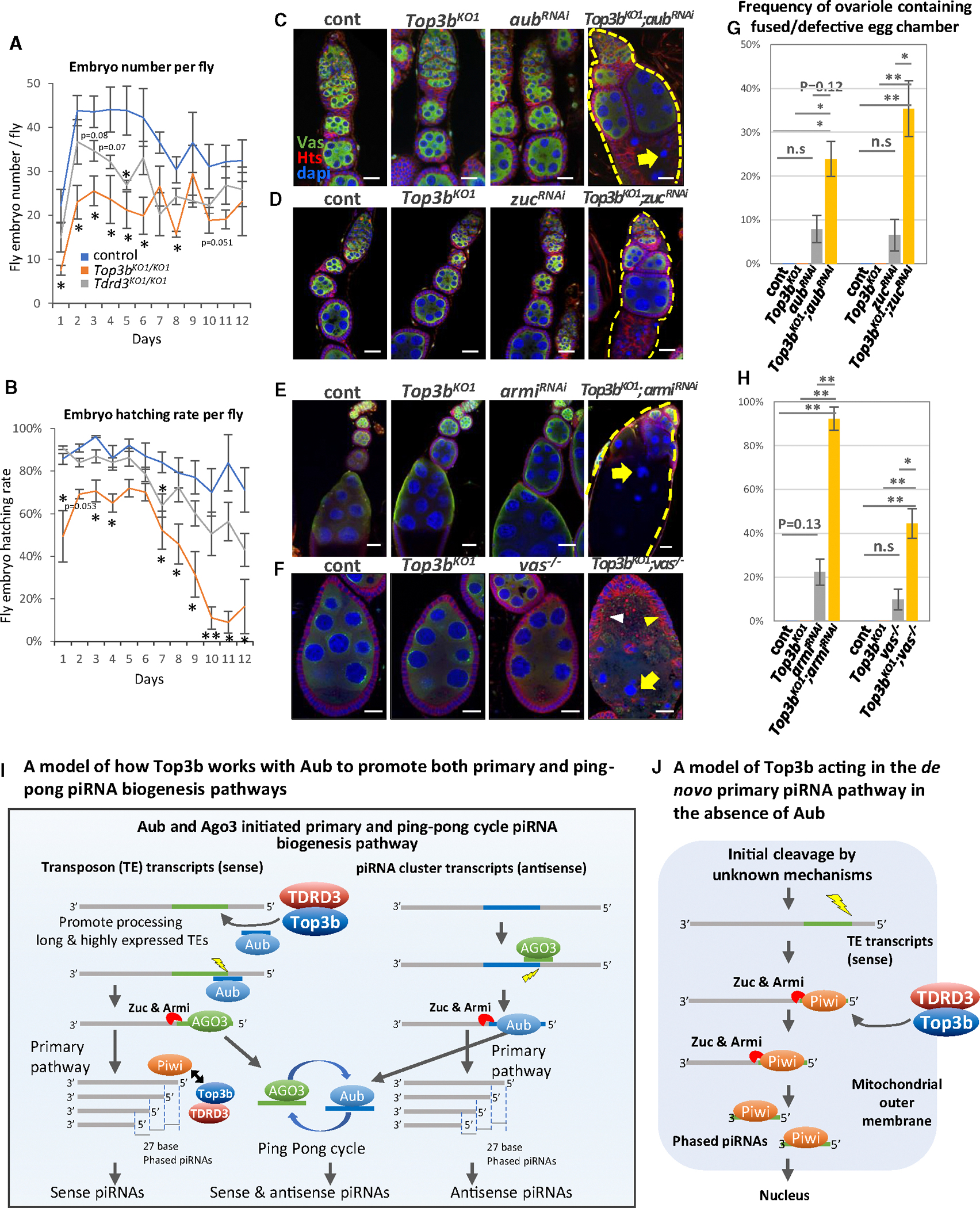
*Top3b* genetically interacts with piRNA processing enzymes to promote germ cell development (A and B) Fertility assay counting average embryo production (A) and larva hatching rate (B) from a single female fly per day. *Top3b*^*KO1*^ (orange) and *Tdrd3*^*KO1*^ (gray) mutants and *w*^*1118*^ control (blue) were counted. (C–F) Confocal images of ovarioles or egg chambers showing positive genetic interactions between *Top3b* and piRNA biogenesis components. Samples were stained for Vas (green, germlines), Hts (red, membranes), and DAPI (blue, nucleus). (C) Control (*nos-gal4*) and *Top3b*^*KO1*^ (*Top3b*^*KO1*^*;nos-gal4*) show normal oogenesis from 1-week-old ovary. *aub*^*RNAi*^ (*nos*>*aub*^*RNAi*^) displays predominantly normal oogenesis, while *Top3b*^*KO1*^*;aub*^*RNAi*^ exhibits fused germarium with cyst and egg chambers (dashed line), and degenerated nurse cells (arrow). (D) Similar to (C), *Top3b*^*KO1*^*;zuc*^*RNAi*^ exhibit high frequency of oogenesis defects compared to control and single mutant. (E) *Top3b;armi*^*RNAi*^ double mutants often exhibit large fused egg chambers with degenerated nurse cells (arrow). (F) Confocal image of stage 8–9 egg chambers shows *Top3b-vas* genetic interaction. *Top3b;vas* double mutant exhibits higher frequency of enlarged germ cells (arrowheads) and degenerated nurse cell nucleus (arrows) compared to single mutants. All scale bars represent 20 μm. (G and H) Quantification of imaging analysis. The *p* values were calculated by two-tailed t test (*p < 0.05 and **:*p* < 0.01). Data are represented as the mean ± SEM. (I) Model of how Top3b promotes PTGS of TEs and sense piRNA biogenesis in germ cells. First, Top3b/TDRD3 is recruited by Aub to the nuage through interactions between the Tudor domain of TDRD3 and methylated residues of Aub. Second, Top3b preferentially binds long and highly expressed TE transcripts to relieve topological stress, which facilitates antisense-piRNA-guided Aub to slice the TE transcripts to initiate primary and ping-pong biogenesis pathways for sense piRNAs. The long and highly expressed TE transcripts may generate more topological structures, such as knots or supercoils, that topoisomerases resolve. (J) Model explaining Top3b’s role in *de novo* primary piRNA biogenesis pathway, independent of Aub (see the discussion for details).

*Top3b* mutant flies mimic *aub* mutants in de-silencing of germline TEs (Figure S6E), suggesting a potential similarity in impaired germ cell function and development, as seen in various piRNA component mutants. We therefore performed a time-course study on the fertility and fecundity of *Drosophila Top3b* and *Tdrd3* mutants, using age-matched *w*^*1118*^ controls. *Top3b*-KO flies exhibited significant reduction of fertility (*p* < 0.05) at 7 of 12 time points, while *Tdrd3*-KO flies showed reduction at 1 time point (Figure 7A). Similarly, fecundity measured by the larva hatching rate was significantly reduced at 10 of 12 time points for *Top3b*-KO flies and 1 time point for *Tdrd3*-KO flies (Figure 7B). These data suggest Top3b plays a more crucial role in fertility and fecundity than TDRD3. Interestingly, both mutants showed stronger hatching rate reduction in old flies (Figure 7B), resembling human genetic studies linking *Top3b* and *Tdrd3* variants to age at natural menopause and premature menopause.

*Top3b*-KO male mice have more severe fertility defects than females.^39^ We found that *Tdrd3*-null female mice^66^ also showed fertility abnormality (Figures S7A and S7B). Together, the data from flies, mouse, and human argue that the Top3b-TDRD3 complex is essential for normal germ cell functions.

We then examined germ cell development in the ovaries of *Top3b* and *Tdrd3* mutant flies and observed developmental arrest and germ cell degeneration in both (Figures S7C–S7K), suggesting that Top3b-TDRD3 promotes normal germ cell development and prevents degeneration.

### *Top3b* interacts with piRNA processing enzymes to promote germ cell development

We next examined the genetic interactions between *Top3b* and several piRNA processing enzymes (*aub*, *armi*, *zuc*, and *vasa*) in germ cell development. Mutations in these enzymes are known to disrupt germline stem cell (GSC) self-renewal and differentiation.^67,68^ We observed two major oogenesis defects significantly stronger in their double mutants with *Top3b* than in their respective single mutants or WT controls (Figures 7C–7H; *p* < 0.05). The first is fused egg chambers, characterized by multiple chambers abnormally fused into a large structure^69^ (yellow dotted line in Figures 7C–7E). The second is defective germline cell morphology, including smaller and fragmented nuclei (Figures 7E–7H), disrupted and aggregated membranes, and lost cytoplasm, possibly indicating germ cell depletion. Different double mutants exhibited distinct phenotypes: *Top3b*-*aub* and *Top3b*-*zuc* mainly exhibited the first impairment, *Top3b-vasa* mainly exhibited the second, and *Top3b-armi* displayed both. The data suggest that *Top3b* genetically interacts with different piRNA processing enzymes to support germ cell development.

## DISCUSSION

### Top3b-TDRD3 functions in germ cell cytoplasm by a unique RNA-based mechanism

Mutations and variants in the *Top3b-Tdrd3* complex have been linked to germ cell dysfunction, based on studies in human and *Top3b*-KO mice.^37–39,70^ Here, we report that a *Tdrd3*-null mouse line and several *Top3b* or *Tdrd3* mutant *Drosophila* strains also display reduced fertility, suggesting that Top3b-TDRD3 is required for germ cell function across vertebrates and invertebrates. Moreover, *Top3b* and *Tdrd3* mutant flies display developmental delays during oogenesis, suggesting that impaired germ cell development contributes to the fertility defects observed in humans or mice carrying *Top3b-TDRD3* mutations.

While other topoisomerases have been implicated in germ cell function,^5,16,71–77^ Top3b-TDRD3 is distinct in that it is localized primarily in the cytoplasm during oogenesis, implying that RNA, rather than DNA, is its primary substrate.

*Top3b*-KO and *TDRD3*-KO flies share several fertility and germ cell development defects (Figures 7A and 7B; Figure S7) but also exhibit distinct phenotypes, consistent with reports that *Top3b*-KO and *TDRD3*-KO mice or cell lines have both common and unique phenotypes.^11,13,14,31^ These data suggest that TDRD3 has additional functions beyond stimulating topoisomerase activity of Top3b, potentially involving interactions with multiple RBPs and RNA polymerase II (RNAPII) through its Tudor domains.^78^

### Top3b works with Aub to initiate sense piRNA biogenesis from TE transcripts

In germ cells, Aub and Ago3 slice different precursor transcripts to initiate sense and antisense piRNA production, respectively^48,65^ (Figure 7I). Aub, guided by antisense piRNAs, cleaves the TE transcripts to initiate sense piRNA biogenesis, whereas Ago3, guided by sense piRNAs, cleaves piRNA cluster transcripts for antisense piRNA production. Our findings that Top3b IP contains Aub but not Ago3 (Figure 1F), and TDRD3 recruitment to the nuage depends on Aub, suggest that Top3b specifically cooperates with Aub to cleave TE transcripts to initiate sense piRNA production (Figure 7I). This model is supported by several pieces of evidence. One, sense piRNA production is more disrupted than antisense in *Top3b* mutants under either a WT or an *aub*-KD background (Figure 6). Two, 10A and 1U piRNA signatures are more reduced on sense than on antisense piRNAs in *Top3b-aub* double mutants (Figures S5G, S5H, S5J, and S5K). Three, Top3b binds TE-derived transcripts, including *burdock*-lacZ (Figure 2B), and several endogenous TEs (Figure S6H). Fourth, Top3b and its topoisomerase activity are required for PTGS but not TGS of the TE transcript from the *burdock*-lacZ reporter.

We observed that the *Top3b-aub* double mutant has stronger disruption of both sense and antisense piRNA biogenesis than their single mutants, though the disruption of antisense is weaker (Figures 6A–6D), suggesting that Top3b can also work with Aub to enhance antisense piRNA biogenesis. One explanation is that *Top3b-aub* double mutation may reduce the initial cleavage of TE transcripts, leading to disruption of both primary and ping-pong pathways downstream (Figure 7I). Because the ping-pong pathway produces both sense and antisense piRNAs, its disruption could explain the reduced antisense piRNA biogenesis.

### Top3b promotes an alternative primary piRNA pathway in the absence of Aub

Our findings that, in the *aub* mutant background, Top3b inactivation strongly disrupts piRNA levels and signatures suggest that Top3b may also act through an alternative mechanism independent of *aub* or ping-pong cycle. Consistent with this notion, our IP-MS and IP-western showed that Top3b-TDRD3 biochemically interacts with not only Aub but also Piwi and Armi (Figures 1F and S2F–S2H), which function in the *de novo* primary piRNA pathway to produce phased piRNAs in both germline and somatic ovary cells^60^ (Figure 7J). This Aub-independent pathway is initiated by an unknown mechanism, not by Aub- or Ago3-mediated cleavage of precursors. We propose that Top3b may help Piwi bind the 5ʹ end of piRNA precursors to establish the cleavage site for Zuc to produce the 3ʹ end of phased piRNAs. In addition, Top3b may cooperate with Armi helicase, which works with Zuc,^79^ to enhance Zuc-dependent cleavage reactions. This model aligns with our data showing stronger distance-dependent decline in the 5ʹ-to-5ʹ phasing signature of total piRNAs in *Top3-aub* double compared to single mutants (Figure S5I), suggesting that the stepwise cleavage reactions that produce phased piRNAs may be attenuated. Moreover, this hypothesis explains how *Top3b* genetically interacts with *piwi* and *zuc*, as well as a somatic piRNA cluster, to silence the somatic *gypsy*-lacZ reporter, reflecting the *de novo* primary pathway’s function in somatic cells. It also explains *Top3b*’s genetic interaction with *armi* and *zuc* in silencing some endogenous TEs.

### Top3b preferentially silences longer and highly expressed TEs

Our findings that the *Top3b* catalytic mutant (*Y332F*) is as defective as *Top3b*-KO in silencing a germline transposon reporter (*burdock*-lacZ) at the mature but not the nascent transcript level suggest that Top3b acts as an RNA topoisomerase to relieve topological stress during PTGS of TE-derived RNAs. Top3b preferentially silences long and highly expressed TEs, mirroring the role of DNA topoisomerases, which favor transcription of long and highly transcribed genes.^14,55^ This suggests that long and highly expressed RNA or DNA may generate more topological problems, such as supercoils, knots, or entanglements, which require topoisomerases to solve (models l and m in Figure S7). Longer RNAs may be harder to relieve of topological stress via rotation than shorter ones, making them more dependent on topoisomerases.

Topoisomerases often work in coordination with helicases to resolve topological stress.^80,81^ We observed biochemical and/or genetic interactions between *Top3b* and several RNA helicases (*vasa* and *armi*) in TE silencing, suggesting that Top3b may work with these helicases to facilitate piRNA biogenesis and PTGS of TEs (Figure 7I). We propose that TE transcripts, which are sense piRNA precursors, may form higher-order RNA structures requiring unwinding by RNA helicases like Vasa or Armi and untangling by Top3b-TDRD3 to enable Aub-piRISC access for sense piRNA production (Figure 7I).

In summary, our data define a key function for a topoisomerase in RNA-based processes—piRNA biogenesis and PTGS of TEs in the cytoplasm. This function could be critical for Top3b-TDRD3 to promote germ cell function across animal species.

### Limitations of the study

We have shown that Top3b preferentially silences long and highly expressed TE transcripts. However, whether these TE transcripts share additional features recognized by Top3b remains unclear. We hope to address this challenge in future studies.

Our analysis for the 10A signature in Ago3-bound piRNAs relied on alignment of our sRNA-seq data with previously published Ago3-bound piRNAs.^60^ A more precise approach would be to immunoprecipitate Ago3-bound piRNAs from WT and mutant flies and directly assess the difference in their 10A signature.

While our study focused on the molecular mechanisms of Top3b in piRNA biogenesis and TE silencing, we did not extensively characterize TDRD3’s role in these processes. Because TDRD3 is a critical regulatory subunit of the Top3b-TDRD3 complex, its roles require an independent investigation in the future.

Our data imply that Top3b may interact with Piwi and Armi to promote a *de novo* primary piRNA biogenesis pathway independent of Aub. This hypothesis requires rigorous validation.

## RESOURCE AVAILABILITY

### Lead contact

Requests for further information and resources should be directed to and will be fulfilled by the lead contact, Weidong Wang (wangw@grc.nia.nih.gov).

### Materials availability

All reagents generated in this study are available from the lead contact with a completed materials transfer agreement.

### Data and code availability

All next-generation sequencing data have been deposited at GEO: GSE230675. Source code used in this study has been deposited with Zenodo: https://doi.org/10.5281/zenodo.14872044.

## STAR★METHODS

### EXPERIMENTAL MODEL AND STUDY PARTICIPANT DETAILS

#### *Drosophila* husbandry and experimental conditions

All flies used in this study were cultured on corn syrup-soy recipe food with yeast granules from Archon Scientific at 25±1°C and 60±5% humidity, under a 12h/12hr light/dark cycle. For control, we used *w*^*1118*^ strain or *nanos-gal4* to match the genetic background. All strains used are listed in the key resources table. For all experiments, age-matched 5–7 days post-eclosion females were used for ovary isolation.

#### Animal studies

Tdrd3-KO and WT C57BL/6N mice^31^ were used for fertility assay. All the animals were housed in temperature-controlled room with 12/12 light/dark cycle. For mouse pup counting, 3–4 month old male (average weight 28.9g) and female (avg weight 22.9g) mice were used for the assay. All animal procedures were approved by the NIA animal care and use committee (ACUC) and followed the NIH animal guidelines (protocol number: 436-LGG-2024).

### METHOD DETAILS

#### TE reporter assay

For the *gypsy-lacZ* and *burdock-lacZ* reporter assays, 2–6 days old ovaries were dissected in cold PBS and fixed in 2% paraformaldehyde and 0.2% glutaraldehyde in PBS at room temperature for 5 min followed by washing with PBS for 3 times. The samples were stained in X-gal solution (0.27%) at 37°*C* for 1–2 hours (*gypsy-lacZ* reporter) or overnight (*burdock-lacZ* reporter). Staining was stopped by PBS wash (10minutes for 3 times at room temperature). Stained samples were mounted and imaged with Leica M165FC microscope. The assay was repeated three times and at least 10 ovaries per sample were analyzed each time.

#### Fertility assay

For fly embryo counting, 20 female virgin flies and 10 male flies were set in a cage with grape juice plate with thin layer of yeast paste. The flies were incubated at 25°C 12h/12h light/dark cycle. The plates were collected every 24 hours to record embryo production. The same plates were checked for the larva hatching after another 24 hours. The experiments were performed minimum 3 times per each genotype.

For mouse pup counting, 3–4 month old male (average weight 28.9g) and female (avg weight 22.9g) mice were used for the assay. Four pairs of 1:1 cross of WT male X WT female, WT male X Tdrd3 KO female, Tdrd3 KO male X WT female were set, and monitored pregnancy and pup production every day for 6 month.

#### Cell culture

OSS cells, stock#190 from *Drosophila* Genomics Research Center (DGRC), were cultured in OSS medium, Shields and Sang M3 Insect Medium (Sigma-Aldrich, S3652–1L) supplemented with 10% heat-inactivated fetal calf serum, 10% fly extract (*Drosophila* Genomics Research Center), 2% Antibiotic-Antimycotic Solution, 1% Glutamax, 1% Glutathione (Sigma-Aldrich G6013–25G), 1% of penicillin and streptomycin (Invitrogen, Thermo Fisher, Waltham, MA USA) and 0.05% Insulin Solution (Sigma-Aldrich I9278–5ml) at 25°C, 5% CO_2_.

#### Immunoprecipitation and Western blot

The OSS cell lysates were extracted using Ripa buffer (Sigma R0278) with proteinase inhibitor. The cell lysates were incubated with anti–Flag M2 agarose beads (A2220, Sigma-Aldrich) or protein A beads with specific antibodies to immunoprecipitate Top3b,TDRD3 and its interacting proteins. Each IP reaction was brought up to 1ml with IP buffer (20mM Hepes pH 7.9, 200mM NaCl, 0.1% NP40) and incubated for 4 hours at 4 C. Wash the beads with IP buffer three times, 5 minutes each time. The immunoprecipitated eluates were analyzed by standard Western blot analysis. For Western blot, a-rabbit fly Top3b and a-rabbit fly TDRD3 was used in 1:3000 dilutions. a-mouse FLAG-M2 (Sigma F3165), Mouse Tdrd3 (Cell Signaling 5942S) and Miwi (Cell Signaling 2079S) were used in 1:1000, 1:1000 and 1:1000, respectively.

#### Mass spectrometry

Mass spectrometry was done by the Harvard Medical School Taplin Mass Spectrometry Facility.

#### RNA-sequencing

A total 50μl of *Drosophila* ovaries was collected by dissecting in cold PBS. Total RNA was extracted using TRIzol reagent according to the manufacturer’s protocol (Thermo Fisher, Waltham, MA USA), followed by cDNA synthesis using SuperScript double-stranded cDNA synthesis kit (Invitrogen 11917–010). Double-stranded cDNA was sonicated using Bioruptor for 4 times 10 min each time with medium power setting (15 sec on/off). Library generation was carried out by standard protocols: 40ul of sonicated cDNA were incubated with 10X End repair buffer (6ul), 2.5mM dNTPs (6ul), 10mM ATP (6ul), and End-repair enzyme mix (1.2ul, Invitrogen IVGN2504) at room temperature for 45min. The samples were purified by MinElute Reaction Cleanup kit (Qiagen 28204) and incubated for 30 min in water bath at 37°C.

#### Small RNA sequencing

Total RNA extracted from 50 pairs of ovaries was run on 15% Tris-urea gel, and 10–50 nt RNA was extracted by cutting and overnight RNA extraction buffer (300mM sodium acetate, 1mM EDTA and 0.25% SDS), followed by ethanol precipitation. The pellet was resuspended with 10ul Qiagen EB buffer. The RNA Library prep was carried out by NEBNext Multiplex Small RNA Library Prep kit for Illumina (NEB#E7300). Minimum three sets of independently prepared RNA samples were applied for RNA and small RNA sequencing.

#### RT-qPCR

100ng of total RNAs was reverse transcribed using TaqMan MicroRNA Reverse Transcription Kit (Thermo Fisher Scientific 4366597) with random hexamer primer (Invitrogen 100026484) in total 20ul reaction. For qPCR, 8ul of the 5X diluted cDNA was mixed with 10ul 2X SYBR Green PCR Master Mix and 2ul of 10uM primer mix and analyzed using ABI7800HT. Given the reaction was run in triplicate, the threshold values (Ct) were converted to fold change difference in standard delta Ct method. The primers used in the analysis are in Table S7.

#### RNA-seq analysis

RNA-seq and small RNA-seq libraries were sequenced by Ilumina Novaseq6000 at 62 bases or Nextseq2000 at 50 bases. RNA-seq analysis was proceeded by genome mapping on dm6 version *Drosophila* standard genome. Reads were also independently mapped to both strands of consensus transposon sequences obtained from FlyBase. To avoid double counting of reads that align on TEs, the Ensembl GTF annotations that were used for gene/transcript quantification were filtered to remove TEs. BAM files for the TE mapping were transformed to BED format and non-perfect mappings were excluded. TE counts were defined as the number of reads aligning on each sequence. TE counts and gene counts were then combined on a single file and were used for further processing and normalization. DEseq2 was used for differential expression analysis. Cutoffs of p-adj<=0.05, |log2fd|>=0.58 were used.

#### piRNA-seq analysis

Reads from small RNA libraries were trimmed at their 3ʹ ends for 30 nucleotides to remove sequencing adaptors using in-house scripts. Subsequently, reads were aligned to consensus transposon sequences using STAR (2.7.10a) as previously described^82^. The following parameters were used for alignment: outFilterMultimapScoreRange 0, alignIntronMax 1, seedSearchStartLmax 20, outFilterMatchNmin 15, and outFilterMatchNminOverLread 0.8. The consensus sequences for transposable elements were downloaded from FlyBase (v9.42). Small RNA expression per transposon was quantified as piRNAs per kilobase (pkb), corresponding to number of aligned reads per transposon normalized by transposon length and multiplied by 1000. Distances between small RNAs were calculated using all possible alignments of all genome-matching reads, taking into account the number of times a small RNA sequence occurs in the library divided by the number of locations where this small RNA maps in the genome (i.e., multi-mapping reads were apportioned). *Z*_0_ scores for phasing were calculated as described in Han et al., 2015 Science^60^, using distances from −10 to −1 and from 1 to 50 as background. *Z*_10_ scores for ping-pong were calculated as described in ref.^61^ using distances from 1 to 9 and from 11 to 20 as background.

For sense vs. antisense piRNA and 1U10A signature analysis, piRNAs with at least 5 read counts were used. The piRNA read counts were separated into sense or antisense by mapping to the sense or antisense strand of the consensus TEs, in a similar way as mapping to both strands of TEs (see above). Differential expression (DE) analysis between mutants and WT were subsequently performed as described for analysis of piRNAs mapped to both strands of TEs. For 1U counting, if the piRNAs has 1U base with a minimum of 5 counts for the sample, it has been counted as 1, otherwise counted as 0. The same selector was also used for 10A counting. The sum of each selector was obtained for unique piRNAs number, unique piRNA with 1U, and unique piRNA with 10A. The percentage of 1U (or 10A) was calculated by dividing the sum of piRNAs with 1U (or 10A) over the sum of total piRNAs.

For overlapping our piRNA reads with the published data of Aub-IP, Ago3-IP, and Piwi-IP, we used RIP-seq data from Han et al, 2015 *Science*^60^ for the analysis. We noted that there is strong overlap among piRNAs immunoprecipitated by antibodies against different PIWI-clade proteins in this study (Figure S5D). To create lists of piRNAs that are uniquely bound by each PIWI-clade protein, we followed the protocol similar to the one described in the paper by Wang et al^61^, and used a cutoff of 3-fold enrichment of piRNA read counts in one IP over those of the other two IPs. This allowed us to assign three non-overlapping groups of piRNAs, with each of which corresponds to piRNAs uniquely bound by Aub, Ago3, or Piwi (Figure S5E). The piRNA reads for each sample and strand over 5 were counted, and tested for overlapping with the IP data of each PIWI-clade protein.

#### Immunostaining

The ovaries were dissected in cold PBS. The samples were fixed in 4% paraformaldehyde (pH 7.6) for 20 min at room temperature. After washing four times, the samples were incubated in 0.1% Triton X100 and 1% normal goat serum in 1X PBS for 30 min at room temperature for permeabilization. The primary antibodies (α-rat Vasa - DSHB 1:100; α-mouse Hts 1B1– DSHB 1:100; α-mouse FLAG m2 – Sigma 1:100; α-rabbit Top3b and Tdrd3 – 1:1000; α-rabbit Armi C-terminal peptides – 1:1000, a kind gift from Dr. W. Theuerkauf) and secondary antibodies (1:400) were incubated in 0.1% Triton X100 and 1% NGS overnight at 4°C. Washing was performed three times in 10 min intervals after each incubation with 0.1% Triton X100 and 1% NGS. Preparations were examined with Zeiss LSM-710 and −880 confocal laser scanning microscopy.

#### RNA immunoprecipitation

Between 100–200μL of *Drosophila* ovaries were harvested and homogenized in 1.5mL of 0.25M sucrose lysis buffer (25mM HEPES, 250mM sucrose, 1mM MgCl2, 150mM NaCl, 0.1% TX100) with fresh 1M DTT (1ul), cOmplete Mini protease inhibitor (Roche,1ul), and RNAse inhibitor (1ul, with an extra 1μL before incubation). After homogenizing, the samples were spun down at 15,000rpm for 10 minutes at 4°C. While centrifuging, 60 μL of Anti-FLAG M2 magnetic beads (Sigma) were washed 3 times with PBS 0.1% TX100, by centrifuging at 3000rpm for 30 seconds each time. The samples were incubated with the FLAG M2 antibody (Sigma) for 4 hours at 4°C in a rotator, then spun down at 3000rpm for 30 seconds. The sample-bound beads were washed 5 times with 1mL each of the 0.25M sucrose lysis buffer with DTT and protease/RNAse inhibitor. Each wash involved a 4°C rotation for 3–5 minutes and spinning at 3000rpm for 30 seconds. After the fifth wash, 750 μL Trizol was added to the beads and the samples were incubated at room temperature for 5 minutes. The samples were spun at 10,000rpm for 2 minutes and the supernatant was collected and transferred to a new tube with 200 μL chloroform added.

For RIP-qRT-PCR analysis to determine whether Top3b binds *burdock*-lacZ transcript, we isolated Flag-Top3b-bound RNAs by RNA-immunoprecipitation (RIP) in ovary lysates from *Flag-Top3b/Top3b*^*KO1*^ heterozygous mutant flies carrying the *burdock*-LacZ reporter, followed by RT-qPCR quantification of the reporter RNA (Figure 2E). A control experiment showed that *burdock*-LacZ is de-silenced in *Top3b*^*KO1/*+^ heterozygous mutant by LacZ staining assay (Figure 2B, score 2), suggesting that the reporter RNA should be available for binding. We then calculated the enrichment of *burdock*-LacZ mRNA or *actin* mRNA (a control) by Flag-Top3b RIP vs. those from input lysates.

### QUANTIFICATION AND STATISTICAL ANALYSIS

Data are represented as mean ± standard deviation (SD) (Figures 1, 2, 3, and 6) and standard error of mean (SEM) (Figure 7). Immunofluorescence imaging, RT-qPCR, and fertility assays were performed at least three independent times with minimum 10 female fly samples. RNA and small RNA sequencing were performed at least three independent times, except for *Top3b*^*Y332F*^, *ago3* mutant and *armi*^*RNAi*^, which were done twice. Each sample for sequencing was from 50–100ul volume of ovaries. Statistical differences comparing two samples were analyzed using the two-tailed t-test (*:p<0.05; **:p<0.01;***:p<0.001;****:p<0.0001). For 10A and 1U signatures (Figure S5), pairwise comparison using a generalized linear model (GLM) with a negative binomial distribution was used.

## Supplementary Material

1

2

3

4

5

6

7

8

## Figures and Tables

**KEY RESOURCES TABLE:** 

REAGENT or RESOURCE	SOURCE	IDENTIFIER

Antibodies		

a-mouse FLAG-M2	Sigma-Aldrich	Cat# F3165; RRID:AB_259529
Mouse Tdrd3	Cell Signaling	Cat# 5942S; RRID:AB_2797626
anti-vasa	Developmental Studies Hybridoma Bank	Cat# anti-vasa; RRID:AB_760351
anti-Hts 1B1	Developmental Studies Hybridoma Bank	Cat# 1B1; RRID:AB_528070
anti-Top3b	Lee et al.	N/A
anti-Tdrd3	Lee et al.	N/A
anti-Aub	Nishida et al.	N/A
anti-Armi	Cook et al.	N/A
anti-Yb	Murota et al.	N/A

Chemicals, peptides, and recombinant proteins		

Shields and Sang M3 Insect Medium	Sigma-Aldrich	Cat# S3652-1L
Fly Extract	Drosophila Genomics Resource Center	DGRC Stock 1645670
GlutaMAX	gibco	35050061
Antibiotic-Antimycotic	gibco	15240062
Glutathione	Sigma-Aldrich	G6013-25G
Penicillin-Streptomycin	gibco	15140122
Insulin	Sigma-Aldrich	I9278-5ml
Paraformaldehyde	Santa Cruz Biotechnology	CAS 30525-89-4
Glutaraldehyde	Sigma-Aldrich	G7776-10ML
X-Gal Solution	Thermo Scientific	R0941
TRIzol	Invitrogen	15596026
SuperScript double-stranded cDNA synthesis kit	Invitrogen	11917010
End-Repair Mix	Qiagen	Y9140-LC-L
MinElute Reaction Cleanup kit	Qiagen	28204
TBE-Urea Gels, 15%	Invitrogen	EC6885BOX
Sodium Acetate	Invitrogen	AM9740
EDTA	Invitrogen	AM9260G
Buffer EB	Qiagen	19086
NEBNext Small RNA Library Prep kit	New England Biolabs	E7300
TaqMa MicroRNA Reverse Transcription Kit	applied biosystems	4366597
Random Hexamers	Invitrogen	N8080127
SYBR^™^ Green Universal Master Mix	applied biosystems	4309155
Triton X-100	Sigma-Aldrich	X100-5ML
HEPES	gibco	15630080
MgCl_2_	invitrogen	AM9530G
NaCl	invitrogen	AM9760G
Sucrose	Sigma-Aldrich	S0389-500G
cOmplete Protease Inhibitor Cocktail	Roche	11697498001
RNaseOU Recombinant Ribonuclease Inhibitor	invitrogen	10777019
Anti-FLAG M2 Magnetic Beads	Sigma-Aldrich	M8823-1 ML
Chloroform	Sigma-Aldrich	C2432-25ML

Critical commercial assays		

Mass spectrometry	Taplin Biological Mass Spectrometry Facility	https://taplin.hms.harvard.edu/

Deposited data		

small RNA-seq:w[1] Ago3-IP	SRA	SRA:SRX695573
small RNA-seq:w[1] Aub-IP	SRA	SRA:SRX695572
small RNA-seq:w[1] Piwi-IP	SRA	SRA:SRX695571
Code for Ping Pong and Phase score	Zenodo	Zenodo:14872044
Raw sequencing data used in this study	GEO	GEO:GSE230675

Experimental models: Cell lines		

OSS	Drosophila Genomics Resource Center	DGRC Stock 190

Experimental models: Organisms/strains		

*Top3b*^*KO1*^ (*Top3b*^*26*^)	Wu et al	N/A
*Top3b* ^*KO2*^	This study	N/A
*Tdrd3*^*KO1*^ (*Tdrd3*^*18-22*^)	This study	N/A
*Tdrd3*^*KO2*^ (*Tdrd3*^*2-1*^)	This study	N/A
*Top3b* ^*Y332F*^	This study	N/A
*armi* ^*RNAi*^	Bloomington Drosophila Stock Center	BL31604
*aub* ^*RNAi*^	Bloomington Drosophila Stock Center	BL35201
*AGO3* ^*T2*^	Bloomington Drosophila Stock Center	BL28269
*AGO3* ^*t3*^	Bloomington Drosophila Stock Center	BL28270
*piwi* ^*2*^	Bloomington Drosophila Stock Center	BL43319
*vas* ^*RJ36*^	Bloomington Drosophila Stock Center	BL5011
*armi* ^*1*^	Bloomington Drosophila Stock Center	BL8513
*gypsy-lacZ* reporter	Bloomington Drosophila Stock Center	BL53723
*burdock-lacZ* reporter	Handler et al	N/A

## Highlights

- Topoisomerase 3b binds transposon-derived RNAs and promotes post-transcriptional silencing
- Top3b interacts with piRNA processing enzymes to enhance biogenesis of sense piRNAs
- Top3b promotes both primary and ping-pong pathways of piRNA biogenesis
- Top3b and piRNA processing enzymes together support germ cell functions and development
