# Supplementary material for: Topoisomerase 3b facilitates piRNA biogenesis to promote transposon silencing and germ cell development
Source: Cell Rep. Author manuscript; Available in PMC 2025 May 13. (PMC12070812; doi:10.1016/j.celrep.2025.115495)
Supplement: 1 [file NIHMS2076716-supplement-1.pdf]

Cell Reports, Volume *44*

Supplemental information

Topoisomerase 3b facilitates piRNA biogenesis to promote transposon silencing and germ cell development

Seung Kyu Lee, Weiping Shen, William Wen, Yuyoung Joo, Yutong Xue, Aaron Park, Amy Qiang, Shuaikun Su, Tianyi Zhang, Megan Zhang, Jinshui Fan, Yongqing Zhang, Supriyo De, Ildar Gainetdinov, Alexei Sharov, Manolis Maragkakis, and Weidong Wang

Inventory of Supplemental Information

Supplemental Figures and Legends S1-7

Figure S1. New *Top3b* and *Tdrd3* mutant *Drosophila* lines generated CRISPR-Cas9

Figure S2. Top3b-TDRD3 complex localizes at cytoplasm and associates with piRNA machinery in fly and mouse germ cells

Figure S3. *Top3b* genetically interacts with piRNA machinery to silence the *gypsy- and burdock-lacZ* reporters

Figure S4. *Top3b* genetically interacts with several piRNA pathway components to preferentially silence long and highly expressed TEs

Figure S5. *Top3b-aub* double mutant shows strong disruption of piRNA signatures of primary and secondary pathways

Figure S6. *Top3b* and *aub* genetically interact to enhance biogenesis of piRNAs that are mapped to both strands of TEs

Figure S7. Top3b-Tdrd3 promotes fertility in aging associated manner in mouse and flies, and models of how Top3b promotes piRNA driven TE silencing

Figure S1. New *Top3b* and *Tdrd3* mutant *Drosophila* lines generated CRISPR-Cas9

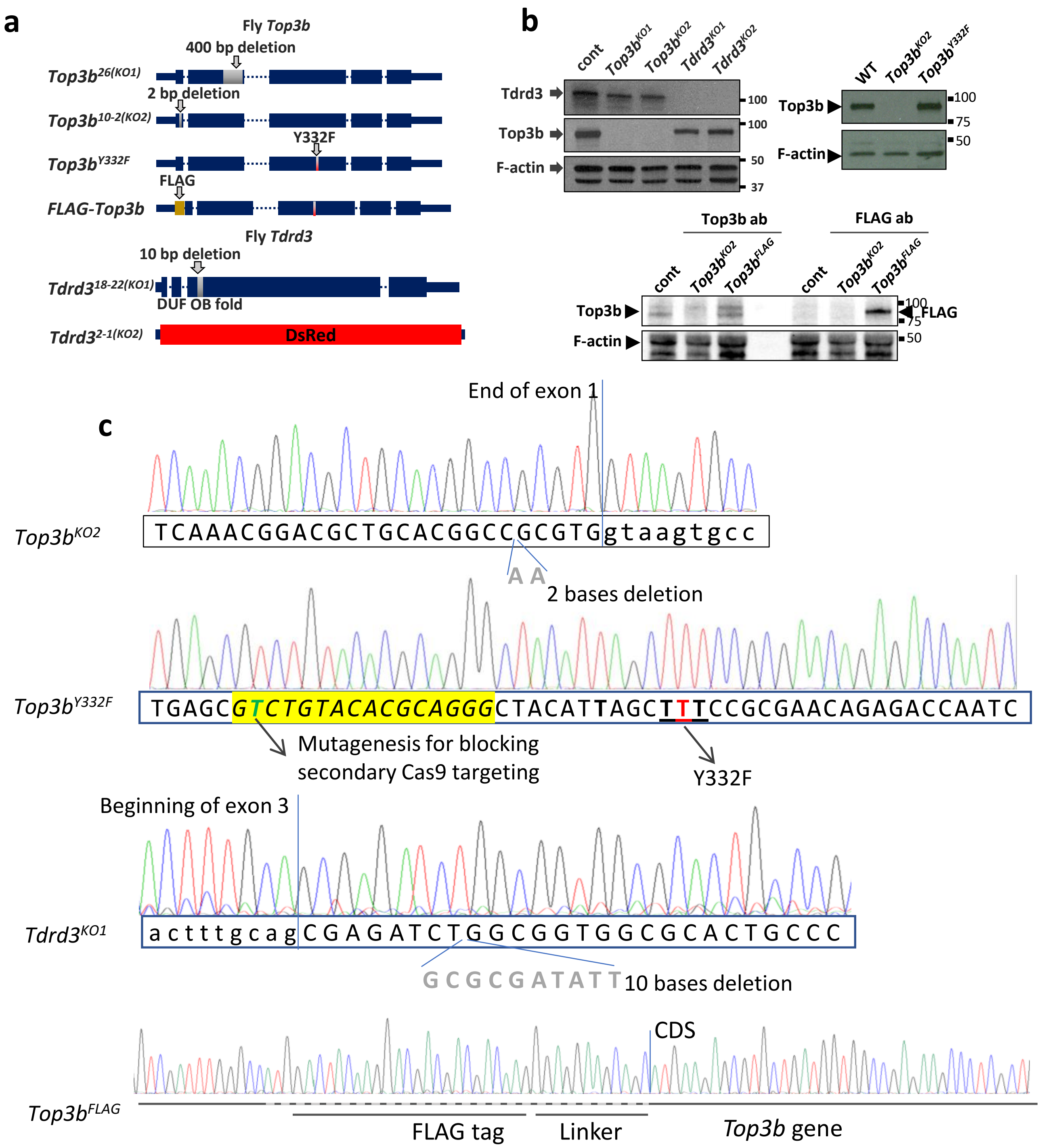

Figure S1. New *Top3b* and *Tdrd3* mutant *Drosophila* lines generated by CRISPR-Cas9. (a) Gene maps of *Top3b* and *Tdrd3* mutant alleles created by CRISPR-Cas9 in this manuscript. *Top3b*^{KO1} (*Top3b*^{26}) was generated by p-element excision by Wu et al[1]. *Top3b*^{KO2} (*Top3b*$^{10\text{-}2}$) was generated by CRISPR-Cas9 indel mutagenesis. *Top3b*^{Y332F} is generated by gene editing using CRISPR knock-in. *Tdrd3*^{KO1} (*Tdrd3*$^{18\text{-}22}$) and *Tdrd3*^{KO2} (*Tdrd3*$^{2\text{-}1}$) were generated by CRISPR-Cas9 indel and gene replacing, respectively. (b) Western blots showing that all mutants are protein null. (c) Sanger sequencing of *Top3b*^{KO1}, *Top3b*^{Y332F}, *Tdrd3*$^{18\text{-}22}$ and *Top3b*^{FLAG} target loci to verify the mutagenesis. Related to Figure 1

Figure S2. Top3b-TDRD3 complex localizes at cytoplasm and associates with piRNA machinery in fly and mouse germ cells

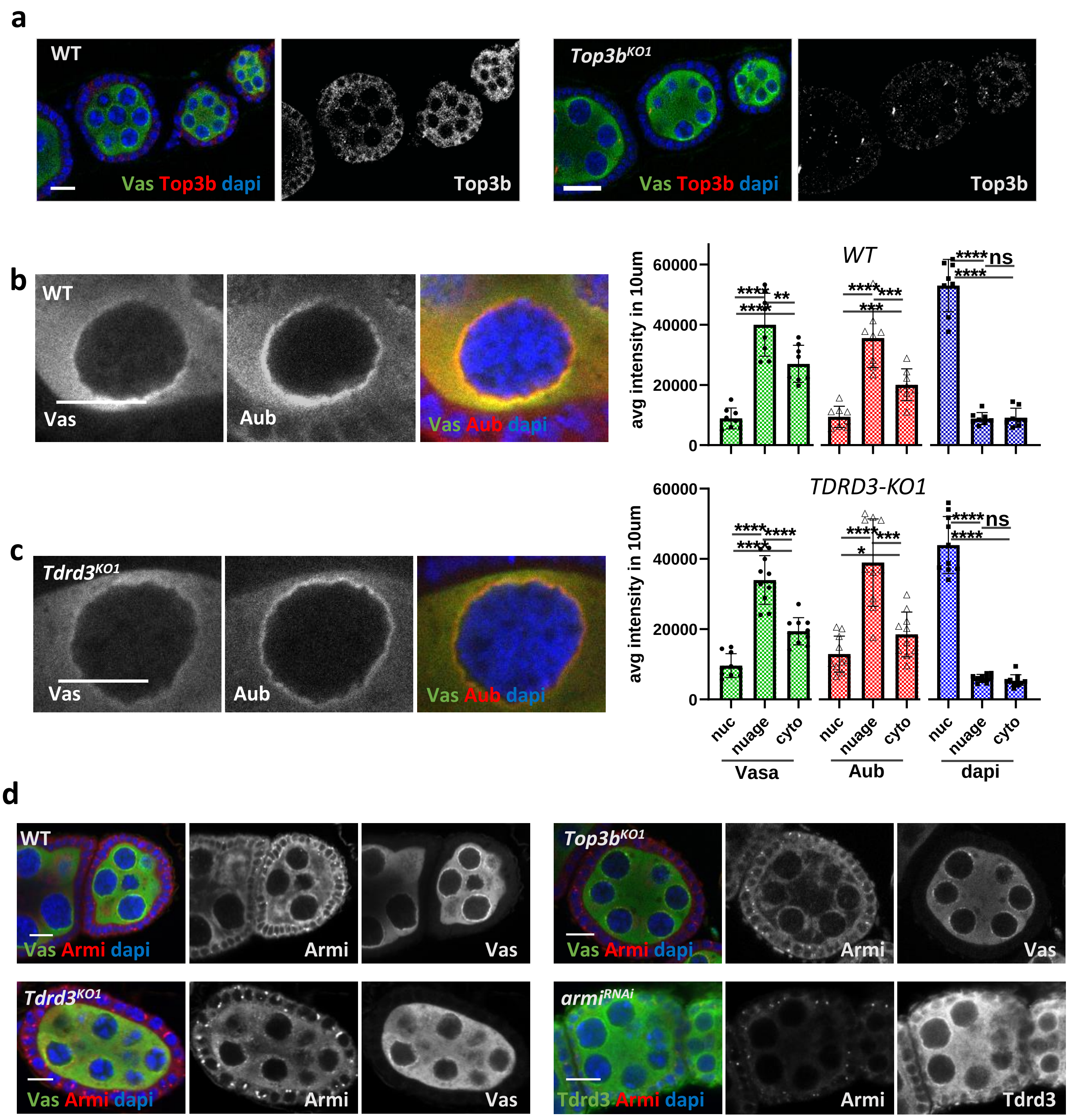

Figure S2. continued

e FLAG Top3b-IPMS GO-term analysis

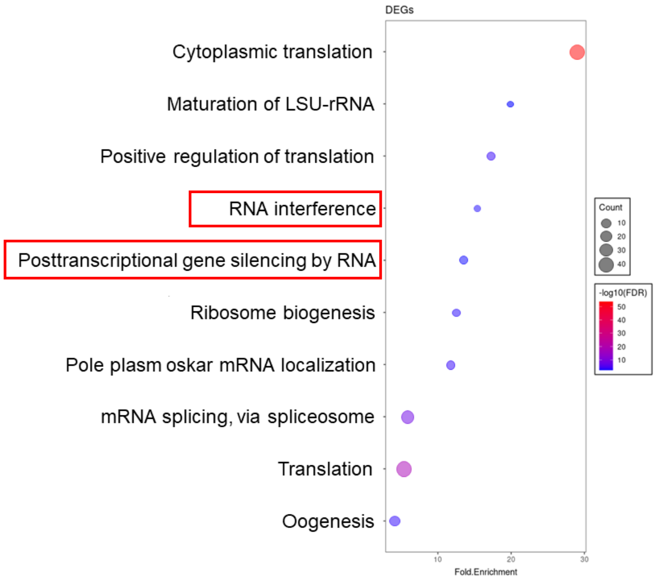

f *Drosophila* OSS cells

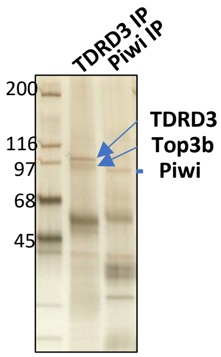

Unique peptide number

	TDRD3 IP-MS	Piwi IP-MS
Tdrd3	65	0
Top3b	52	7
Armi (C)	45	68
PIWI (N)	32	75
Fmr1 (C)	31	15
AGO2	30	36
Rm62	28	23
Bel	19	24
Spn-E (C)	17	29
Dcr-2	14	7
Fs(1)Yb (C)	12	7
Shu	4	6
Nxf2 (N)	4	4
Panx (N)	4	0
Nxf1 (N)	3	2

g *Drosophila* OSS cells

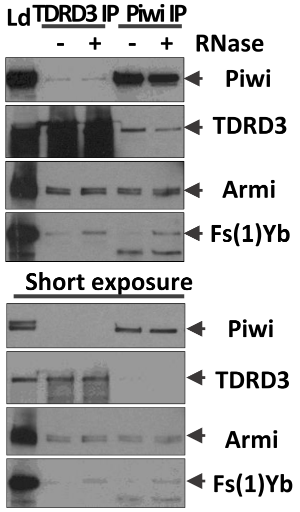

h Mouse testis

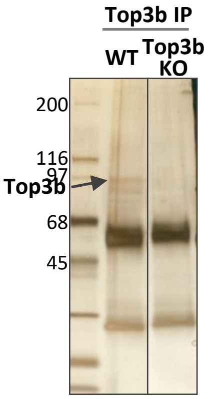

Unique peptide number

	WT	Top3b KO (control)
Top3b	42	0
Tdrd3	30	0
Fxr1 (C)	30	0
Tdrd6 (C)	16	0
Piwil1 (N)	6	0
Fxr2 (C)	6	0
Ddx4 (C)	5	0
Fmr1 (C)	4	0
Mago (N)	4	0
Tdrd9 (C)	3	0

Figure S2. Top3b-TDRD3 complex localizes at cytoplasm and associates with piRNA machinery in fly and mouse germ cells. (a) Confocal image of egg chamber stained with Top3b and Vasa antibodies in w^{1118} control (left) and $Top3b^{KO1}$ (right) (b) Confocal image of control egg chamber stained with Aub (red) and Vas (green) showing co-localization at nuage. The quantification is on the right. (c) Same staining of $Tdrd3^{KO1}$ egg chamber. (d) Confocal images of WT, $Top3b^{KO1}$, $Tdrd3^{KO1}$ and $nos>armi^{RNAi}$ egg chambers stained with Vas (green) and Armi (red) (WT, $Top3b^{KO1}$, and $Tdrd3^{KO1}$), and Tdrd3 (green) and Armi (red) ($nos>armi^{RNAi}$). All scale bars represent 10um. Data are represented as mean +/- SD. Statistical analysis was done by two-tailed t-test (*:p<0.05; **:p<0.01;***:p<0.001;****:p<0.0001) (e) GO-term analysis of fly FLAG-Top3b IP-MS. Red boxes highlight small RNA mediated gene silencing pathways. (f) IP-MS with *Drosophila* OSS cell extract. Silver stain image is on the left. Red represent known piRNA machinery. (g) Scanned Western blot image of TDRD3 IP and Piwi IP with OSS cells. (h) IP-MS with mouse testis extract. Top3b IP was done in WT (left) and *Top3bKO* (right) extract. Related to Figure 1.

Figure S3. *Top3b* genetically interacts with piRNA machinery to silence the *gypsy-* and *burdock-lacZ* reporters

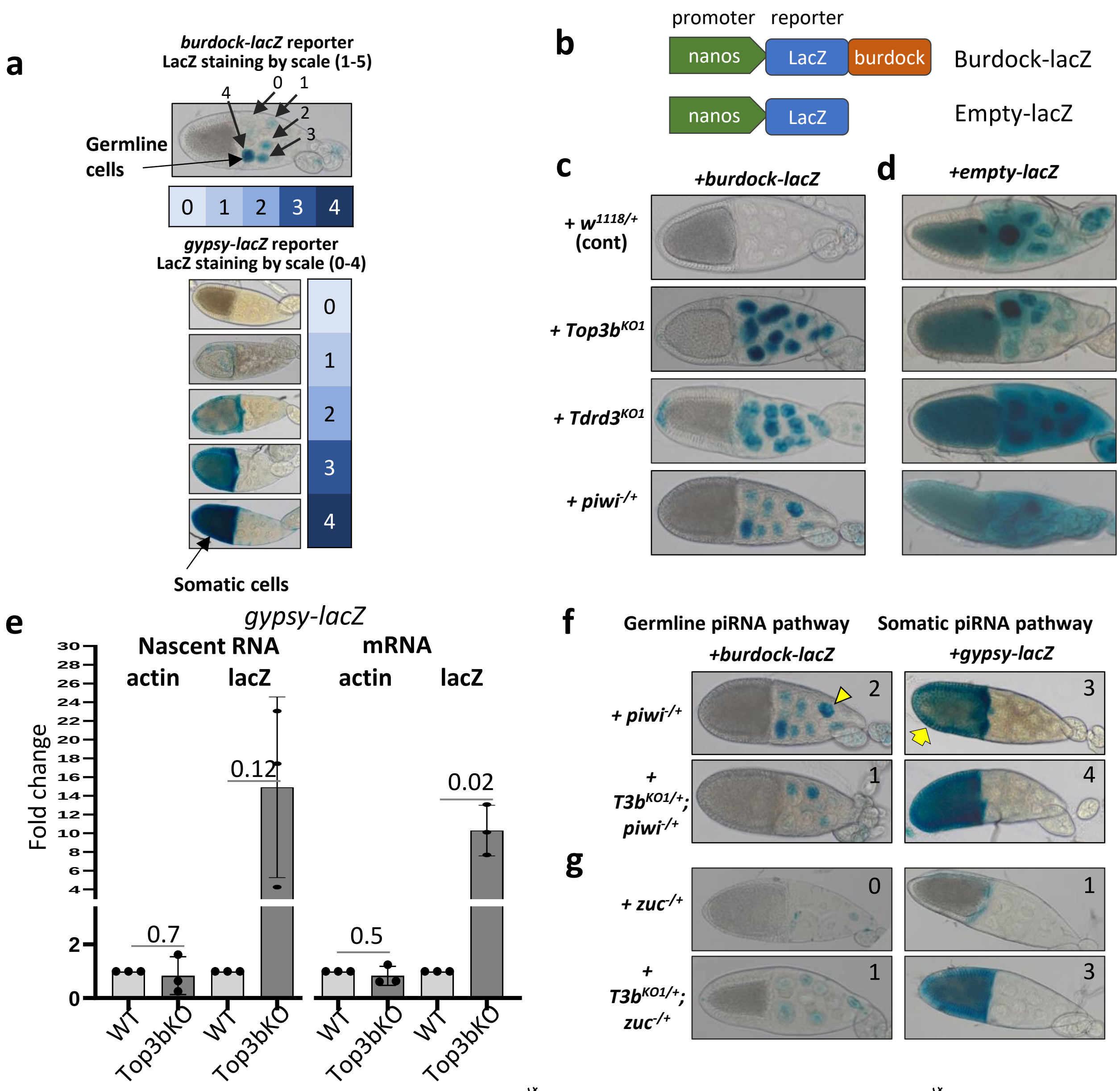

h

Genes tested for genetic interaction by *burdock-lacZ*	*Top3b*$^{KO1/+}$	*mut*$^{/+}$	*Top3b*$^{KO1/+}$;*mut*$^{/+}$	Genetic intxn
Top3b-aub	2	2	2	N
Top3b-ago3	2	1	1	s
Top3b-zuc	2	0	1	s
Top3b-piwi	2	2	1	s

i

Genes tested for genetic interaction by *gypsy-lacZ*	*Top3b*$^{KO1/+}$	*mut*$^{/+}$	*Top3b*$^{KO1/+}$;*mut*$^{/+}$	Genetic intxn
Top3b-piwi	1	3	4	e
Top3b-zuc	1	1	3	e
Top3b-vas	1	1	1	N

j

Genes tested for genetic interaction by *gypsy-lacZ*	*flam*$^{Df/+}$	*mut*$^{/+}$	*flam*$^{Df/+}$;*mut*$^{/+}$	Genetic intxn
Top3b-flam	0	1	4	e
piwi-flam	0	3	3	N
aub-flam	0	0	0	N

k

Genes tested for genetic interaction	*Top3b*^{KO1}	*armi*^{RNAi}	*Top3b*^{KO1};*armi*^{RNAi}	Genetic intxn
Top3b-armi (germline)	4	4	4	N
Top3b-armi (somatic)	4	4	4	N

e: enhancement (positive genetic interaction)
s: suppression (negative genetic interaction)
N: no genetic interaction

Figure S3. continued

Figure S3. *Top3b* genetically interacts with piRNA machinery. (a) Representative LacZ staining scale from *burdock-lacZ* reporter (top) and *gypsy-lacZ* reporter (bottom) (b) Cartoon showing *burdock-lacZ* and control empty lacZ reporter constructs. (c,d) Reporter assays presenting b-gal staining of *burdock-lacZ* reporter (d) and empty-lacZ reporter (d). Note that empty lacZ reporter egg chamber exhibit full expression of lacZ in nurse cells. (e) Bar graph showing the relative expression level of *actin* and *gypsy-lacZ* assayed by RT-qPCR. Left is tested with nascent RNAs and right is with mRNA. (f) Representative images of *burdock*- and *gypsy-lacZ* reporter assays with $piwi^{+/-}$ heterozygous mutant, $Top3b^{+/-};piwi^{+/-}$ double heterozygous mutant. (g) Same as e, but $zuc^{+/-}$ instead of $piwi^{+/-}$. (h) *gypsy-lacZ* reporter assay in *flam Df* background. (h,I,j,k) Summary for genetic interaction assay. For each test (raw), there are 3 colored sections representing b-gal staining intensity: $Top3b^{+/-}$, testing gene mutant, and double mutant (from left to right). The last section determines whether *Top3b* genetically interacts with the tested gene. (k) Genetic interaction test between *Top3b* and *armi*. $armi^{RNAi}$ as a positive control. Quantification table is shown on a. All quantifications are based on minimum 3 independent experiments collecting minimum sample number of 10. Related to Figure 2.

Figure S4. *Top3b* genetically interacts with several piRNA pathway components to preferentially silence long and highly expressed TEs

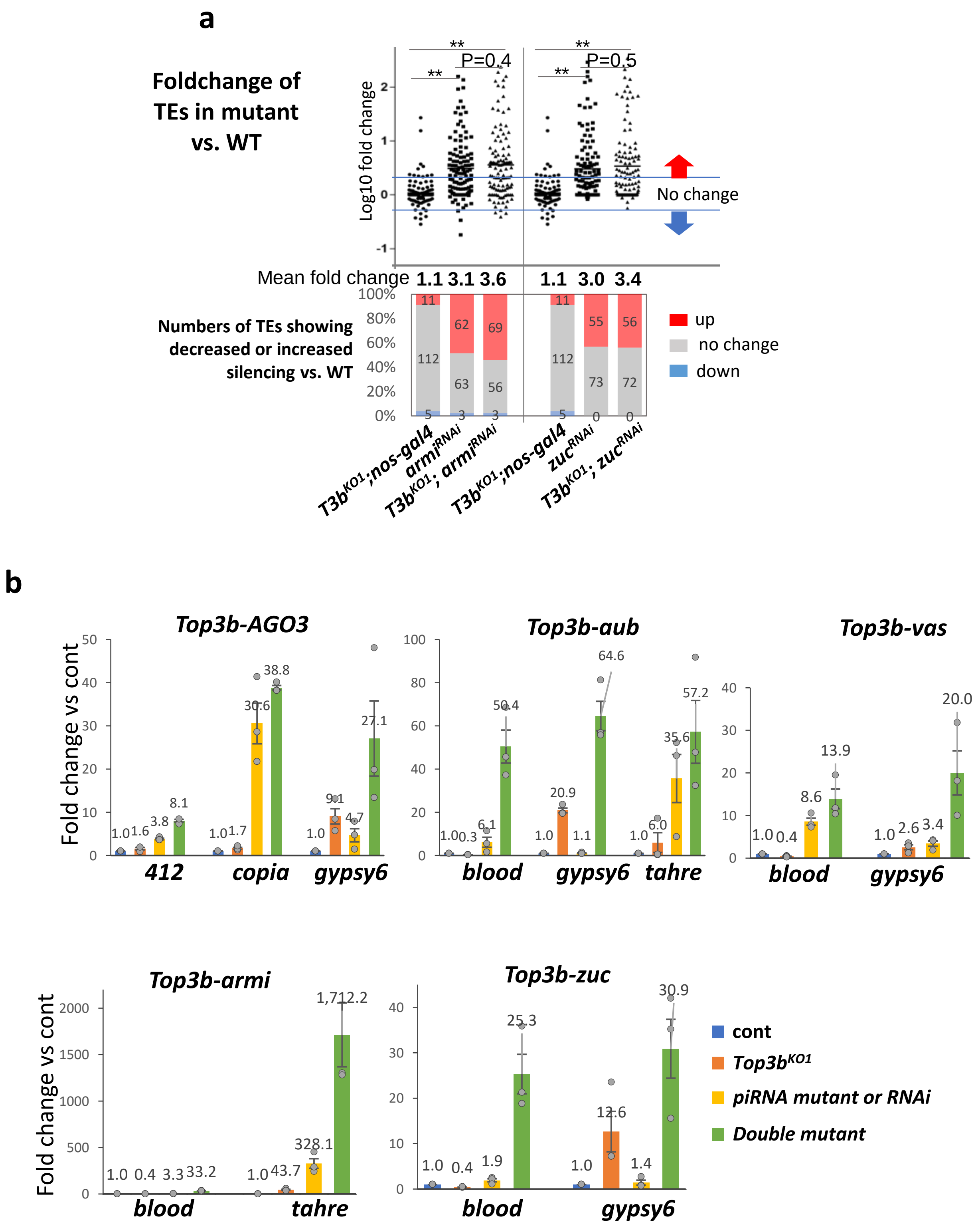

Figure S4. continued

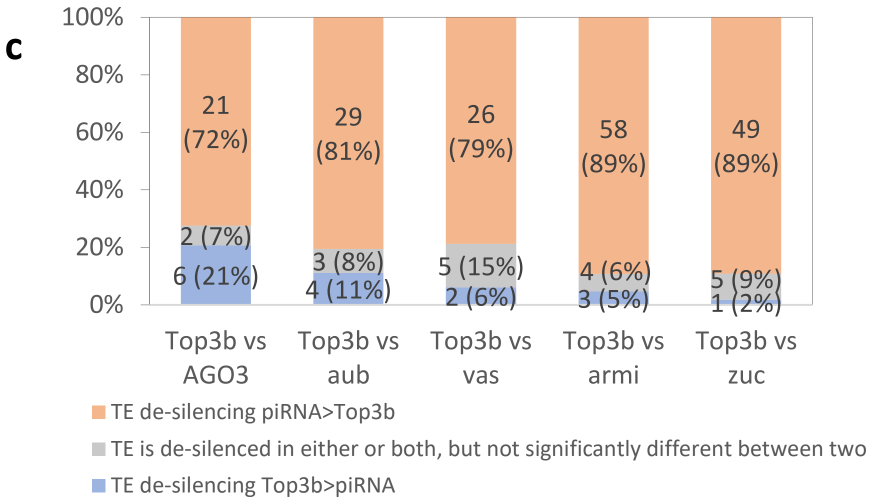

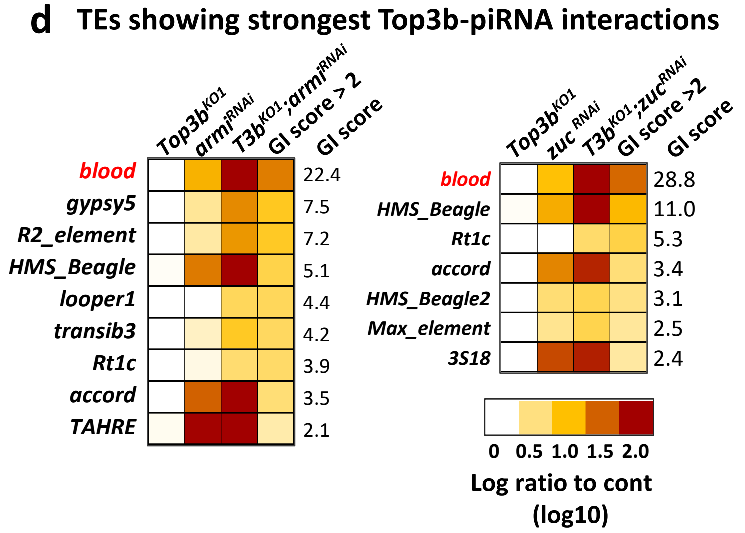

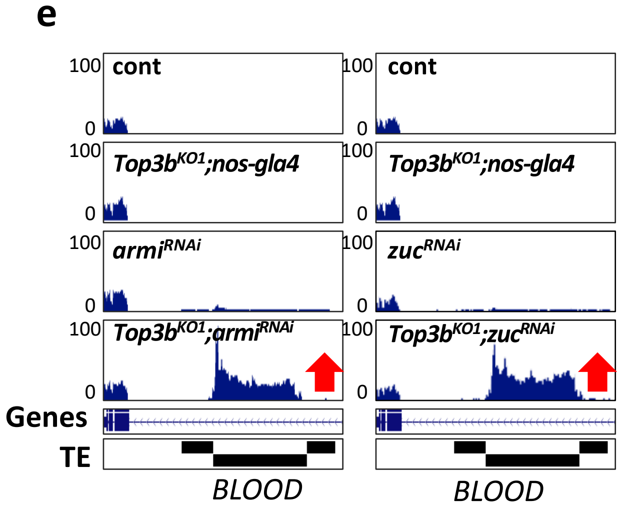

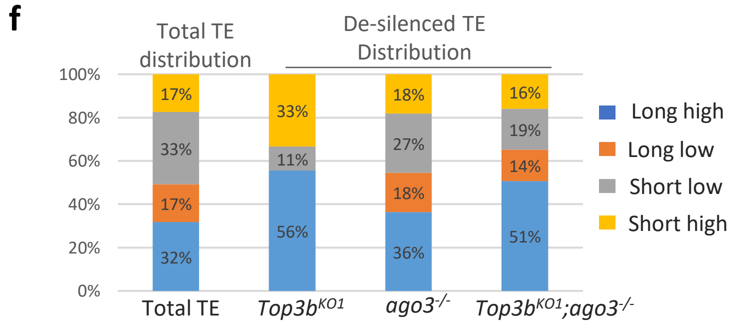

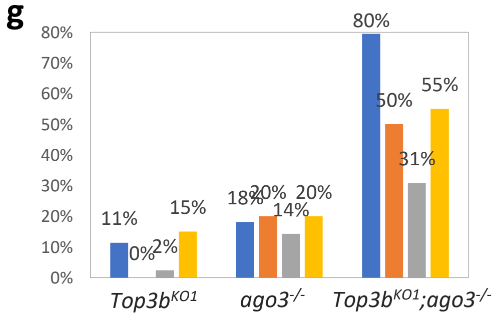

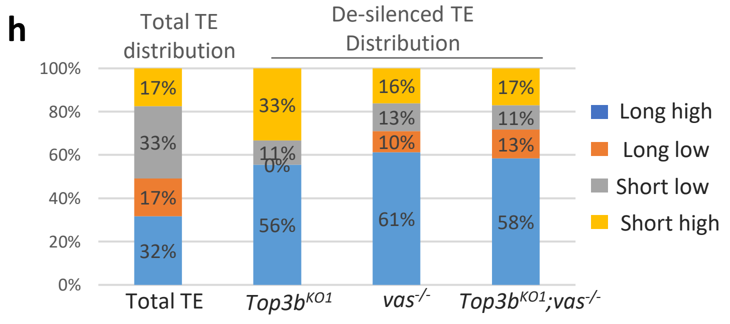

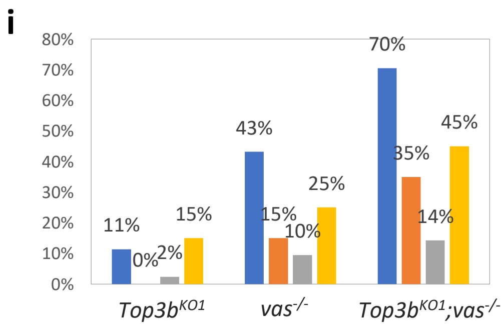

Figure S4. continued

Figure S4. *Top3b* genetically interacts with several piRNA pathway components to preferentially silence long and highly expressed TEs. (a) dot graphs showing expression of TEs compared to control. Bottom column graph show the number of de-silenced (red), no change (gray) or silenced (blue) TEs in each mutant compared to control. Genotypes are labeled on the bottom. (b) RT-qPCR data of the TE RNA levels in w^{1118} control (blue), $Top3b^{KO1}$ (orange), piRNA single mutant (yellow) and *Top3b;*piRNA double mutant (green). The numbers on the graph indicate fold change. The results of RT-qPCRs were from triplicates. (c) Comparison of altered TE number and ratio in single piRNA component mutant vs $Top3b^{KO1}$. Compared mutant genotypes are shown on the bottom. Red indicates TEs significantly higher in piRNA mutant than $Top3b^{KO1}$. Grey indicates no different, and blue indicates TE level is higher in $Top3b^{KO1}$ than piRNA mutant. (d) Heatmap for TE changes, same as Fig. 3d. (e) Genome browser for blood TE locus. Same as Fig. 3e. (f,h) Distribution of desilenced TEs in single or double mutants between *Top3b* and *ago3* or *vasa*. The de-silenced TEs are divided into 4 quadrants based on lengths and expression levels based on the criteria in Figure 4 legend. The 4 quadrants are: Long-high, Long-low, Short-low, Short-high). Color codes are on the right. The cutoffs are average Log10 length 3.7 (NTs) and log10 expression level 1 (rkpm). (g,i) Density of desilenced TEs in each quadrant for the single and double mutants between *Top3b-ago3* (b) and *Top3b-vas* (d). Data are represented as mean +/- SD (S4a) and SEM (S4b). Statistical analysis was done by two-tailed t-test (*:$p<0.05$; **:$p<0.01$;***:$p<0.001$;****:$p<0.0001$). Related to Figure 3.

Figure S5. *Top3b-aub* double mutant shows strong disruption of piRNA signatures of primary and secondary pathways

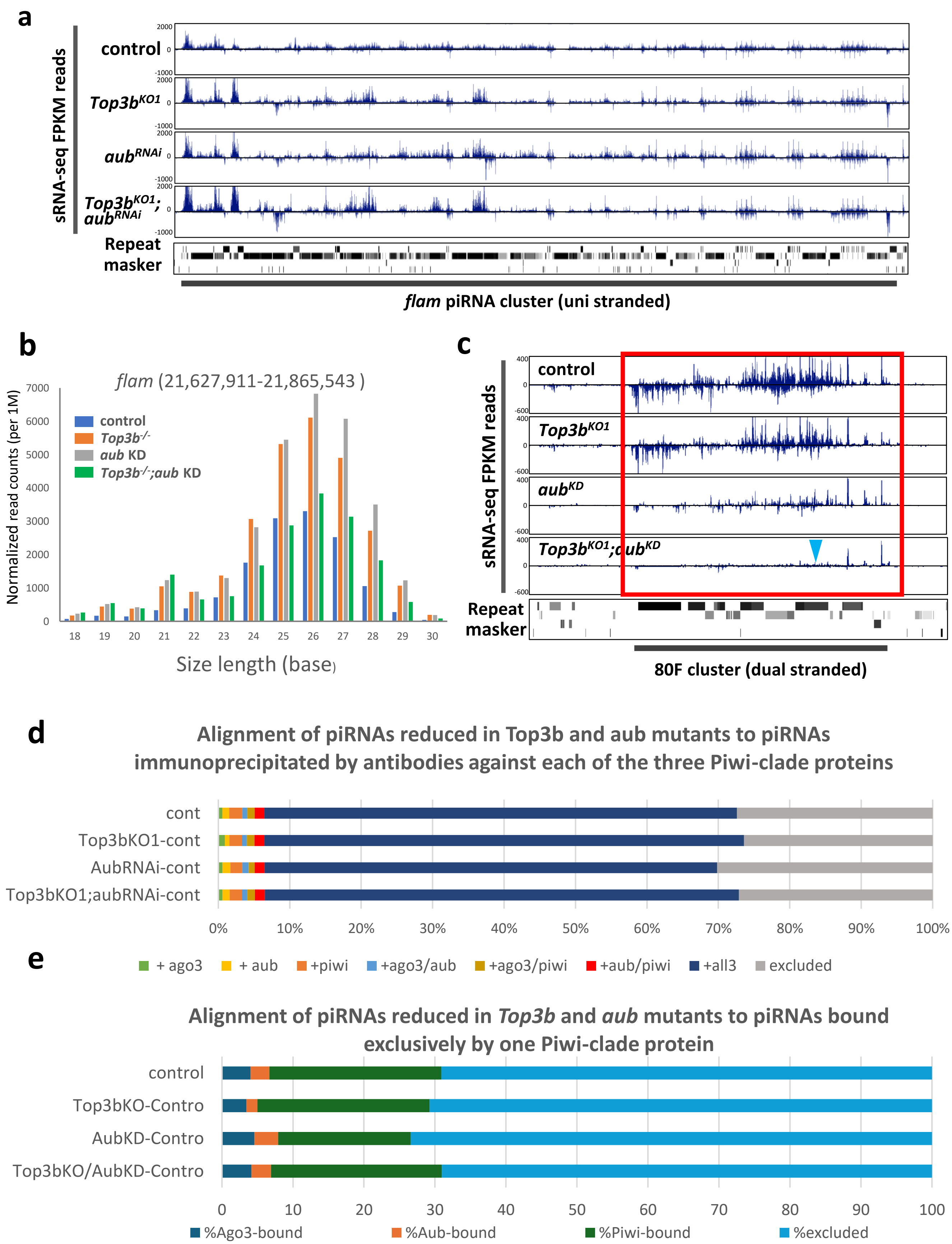

Figure S5. continued

f

10A signature in total piRNAs with cutoff=read counts>5

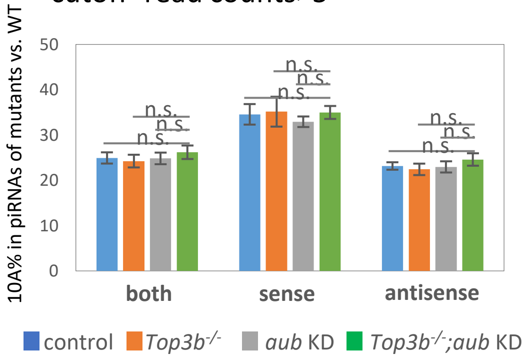

i

3'-to-5' and 5'-to-5' phasing profiles of total unmapped piRNAs in mutants vs. WT

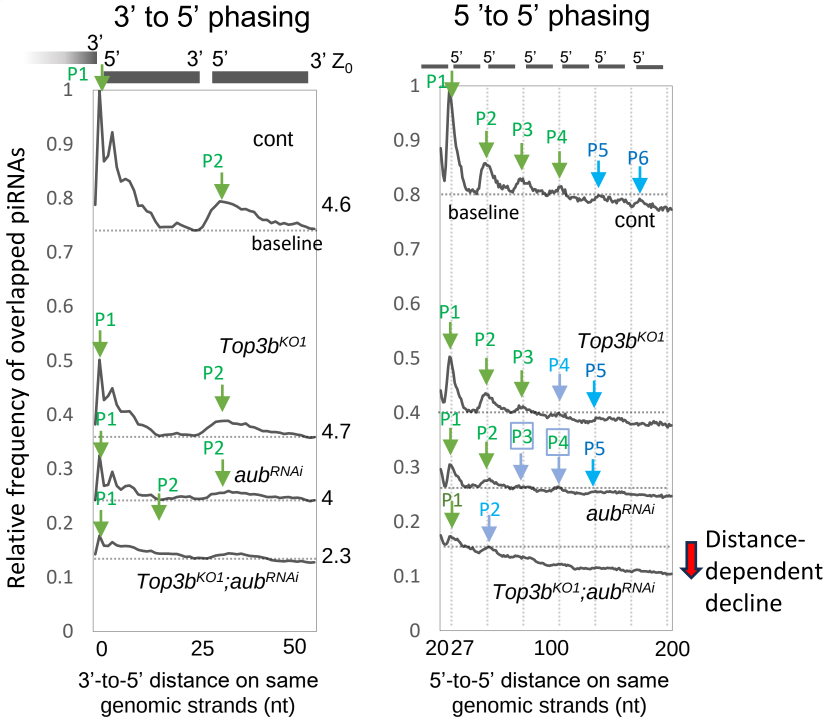

g

10A signature of Ago3-bound sense or antisense piRNAs between mutant vs. WT

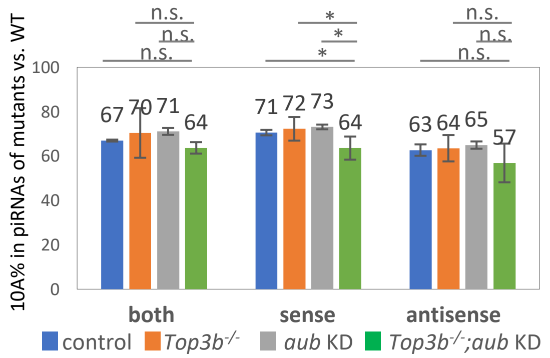

h

10A% difference for Ago3-bound piRNAs between mutants vs. WT

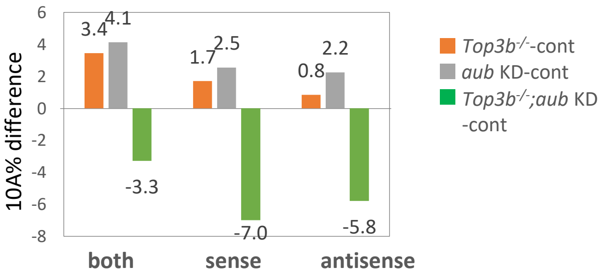

j

1U signature of Total piRNAs of sense or antisense between mutant vs. WT

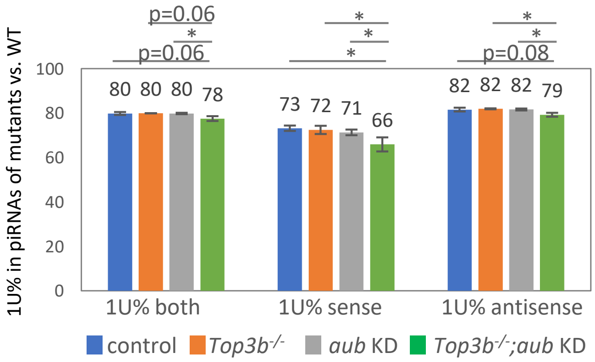

k

1U% difference for total piRNAs between mutants vs. WT

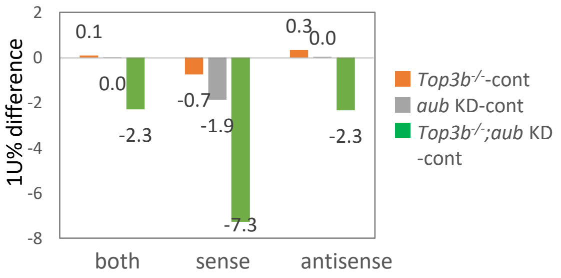

Figure S5. continued

Figure S5. *Top3b-aub* double mutant shows strong disruption of piRNA signatures of primary and secondary pathways. (a) Genome browser view of sRNA sequencing same as Fig 5a but on *flam* locus. Genotypes are shown on left. (b) Size distribution (19-29nt) of the sRNA reads that mapped on *flam* locus from sRNA-seq. (c) Genome browser view of small RNA-seq *80F* cluster. Note that *Top3b;aub*^{RNAi} exhibits very low level of small RNA reads (arrowhead) as compared to other genotypes. (d) A Graph showing the reduced piRNAs in single and double mutants between *Top3b* and *aub* are aligned to the piRNAs immunoprecipitated by antibody against one of the three Piwi-clade proteins (Piwi, Aub or AGO3), or their different combinations. The IP-ed piRNAs from Han et al. Science 2015 were used for the alignment. Note that there is a strong overlap in the piRNAs IPed by the 3 different antibodies. As a result, the majority (~60%) of the piRNAs reduced in each mutant (marked as mutant-cont) or from the WT control were aligned to the overlapped piRNAs IP-ed by all three antibodies. Only a minority of sRNA reads (<30%) cannot be aligned to the piRNAs from the IPs. (e) A Graph showing that the reduced piRNAs in single and double mutants or from WT controls are assigned to piRNAs that are uniquely bound by one of the three Piwi-clade proteins. We used a strategy similar to the one by Han et al. Science 2015) to assign piRNAs to three non-overlapping groups of piRNAs uniquely bound by each Piwi-clade protein. We used a cutoff of 3-fold enrichment of piRNA read counts in one IP over those of the other two IPs to select the group of piRNAs uniquely bound by one Piwi-clade protein. This cutoff excluded nearly 70% of piRNAs in our sRNA-seq samples.

(f) Graph showing 10A% in total piRNA of sense and antisense with cutoff read count 5. (g) A graph showing 10A% in Ago3-bound piRNAs of sense, antisense, or both strands for different mutants and WT. The asterisk marks significant difference ($p<0.05$), whereas n.s. indicates no-significance ($p>0.05$). (h). A graph showing differences in 10A% in Ago3-bound piRNAs of sense, antisense, or both strands for different mutants and WT. (i) Graph showing 3-to-5' and 5'-to 5'- phasing signatures for total unmapped piRNAs. These profiles are similar to those of piRNAs mapped to the 42AB locus shown in Fig. 5e,f. Z_0 is average z score ($p<0.05$) for 3' to 5' distance. The peaks with 27 bp intervals are marked by green arrows. The baseline of each profile is defined as the average value at the valley between Peak 1 and 2. The Blue arrows marked the peaks that are at or below the baseline, which suggest a distance-dependent decline of signals. The red arrow marks a strong distance-dependent decline for the signals at the double mutant. (j,k) Same as *g* and *h* except that it shows 1U% in total piRNAs of sense, antisense, or both strands for different mutants and WT. Related to Figure 5.

Figure S6. *Top3b* and *aub* genetically interact to enhance biogenesis of piRNAs that are mapped to both strands of TEs

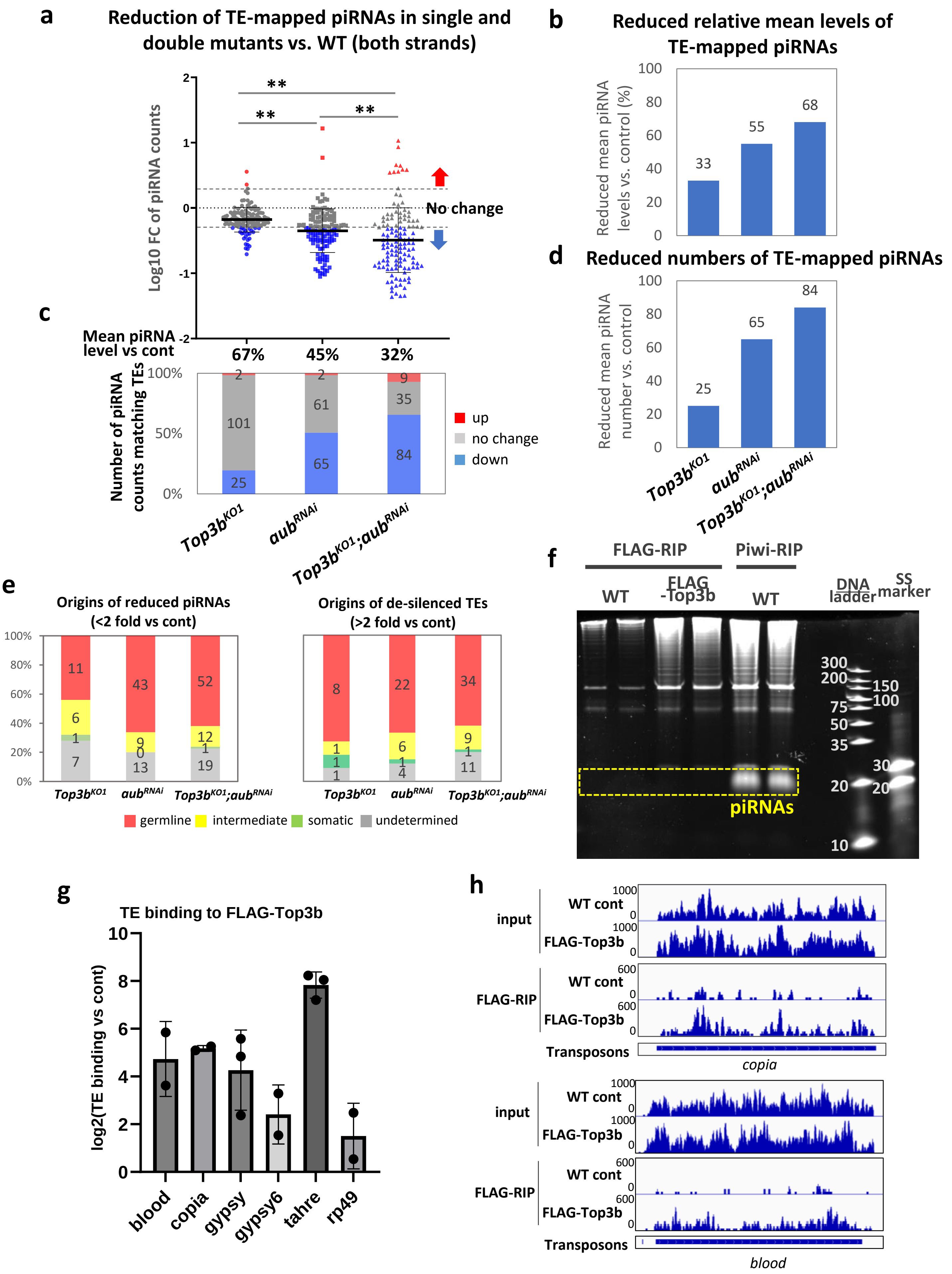

Figure S6. continued

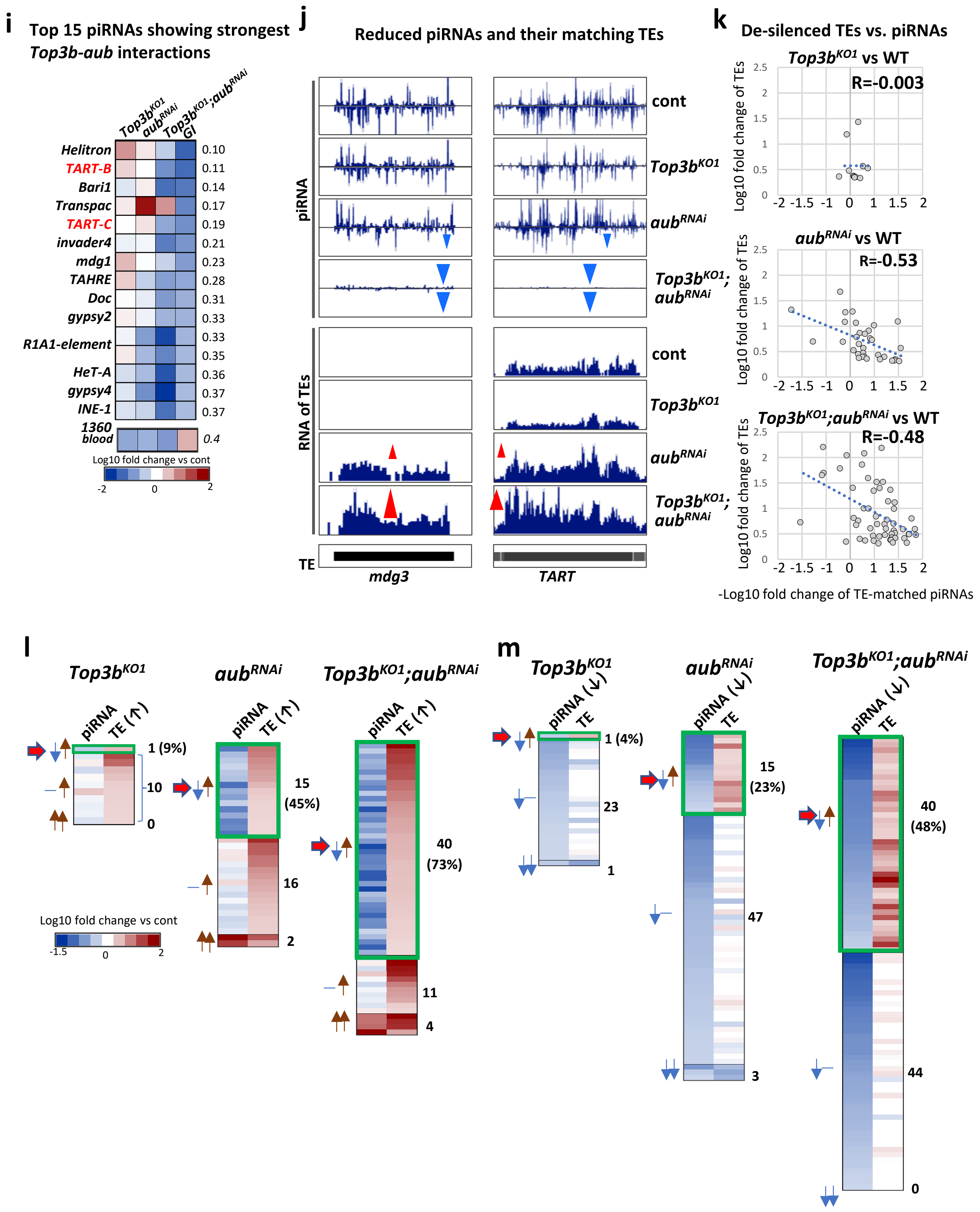

Figure S6. continued

Fig. S6. *Top3b* and *aub* genetically interact to enhance biogenesis of piRNAs that are mapped to both strands of TEs. (a) Whisker plots showing log10 fold-changes of TE-mapped piRNA counts in *Top3b* and aub^{RNAi} single or double mutant. Each data point represents a group of piRNAs mapped to both strands of one TE. The up or down-regulated TE-mapped piRNAs (2-fold cutoff) are marked by red or blue arrows, respectively. The mean relative piRNA levels are listed below, with that of WT as 100%. (b) Graph showing reduction in relative mean levels of sense vs. antisense TE-mapped piRNAs. (c) A graph showing the numbers and percentages of up, unchanged, or down-regulated piRNA-mapped TEs (sense, antisense, or both strands) in single and double mutants vs. WT flies.(d) Graph showing reduced numbers of TE-mapped piRNAs. (f) PAGE gel analysis of RNA-IP samples using Flag antibody from WT (served as a negative control), or Flag-Top3b transgenic fly ovary extracts (left). RNA-IP using Piwi antibody (right, as a positive control) shows a strong small RNA band between 20-30 nt (in box), which are from piRNAs; and this band is absent in Flag-Top3b IP, suggesting that Flag-Top3b does not bind piRNAs. (g) A graph showing RIP-qPCR data to analyze RNAs derived from different TEs bound by Flag-Top3b. RIP-qPCR was performed using a FLAG antibody and lysates from ovaries of FLAG-Top3b flies. Rp49 was included as a normalization control: the levels of different TE RNAs are relative to that of Rp49 RNA. (h) fpkm bedGraph showing input (top) and Flag-Top3b RIP-seq data (bottom) at *copia* and *blood* loci. The genotypes are on the left. WT control flies lack Flag-Top3b and serve as a negative control. (i) Heatmap showing top 15 TE mapped piRNA changes vs control based on sRNA-seq. GI score is on the right. (j) Genome browser view of FPKM bedGraph showing piRNA reads (top 4 panels) on two TE loci, *mdg3* and *TART*. Bottom 4 panels show FPKM bedGraph of RNA-seq that match the same loci. Genotypes are indicated on the right. (k) dot graph shown correlation between de-silenced TE and reduced piRNAs. Correlation coefficient (R) value is shown in the graph. (l-m) Heatmaps shown piRNA (left) and corresponding TE changes (right) based on small RNA-seq and RNA-seq. (l) is clustered and ordered by de-silenced TE (strong to weak, top to bottom), and (m) is clustered and ordered by reduced TE mapped piRNAs. Green boxes represent significant changes of both TEs and piRNAs (2 fold or more). Data are represented as mean +/- SD. Statistical analysis was done by two-tailed t-test (*:$p<0.05$) for qPCR, Pairwise comparison using a generalized linear model (GLM) with a negative binomial distribution for piRNA signatures. Related to Figure 6.

Figure S7. Top3b-Tdrd3 promotes fertility in aging associated manner in mouse and flies, and models of how Top3b promotes piRNA driven TE silencing

a

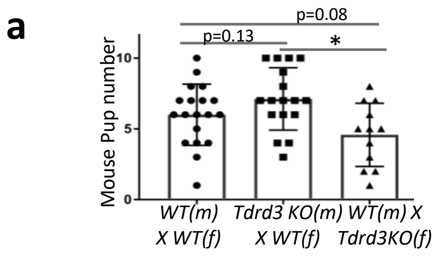

b

Mouse genotype	Total pup number	Total delivery number
WT (m) x WT (f)	115	20
Tdrd3 KO (m) x *WT* (f)	122	17
WT (m) x *Tdrd3* KO (f)	55	12

4 pairs of mice in each cross; 8 month duration

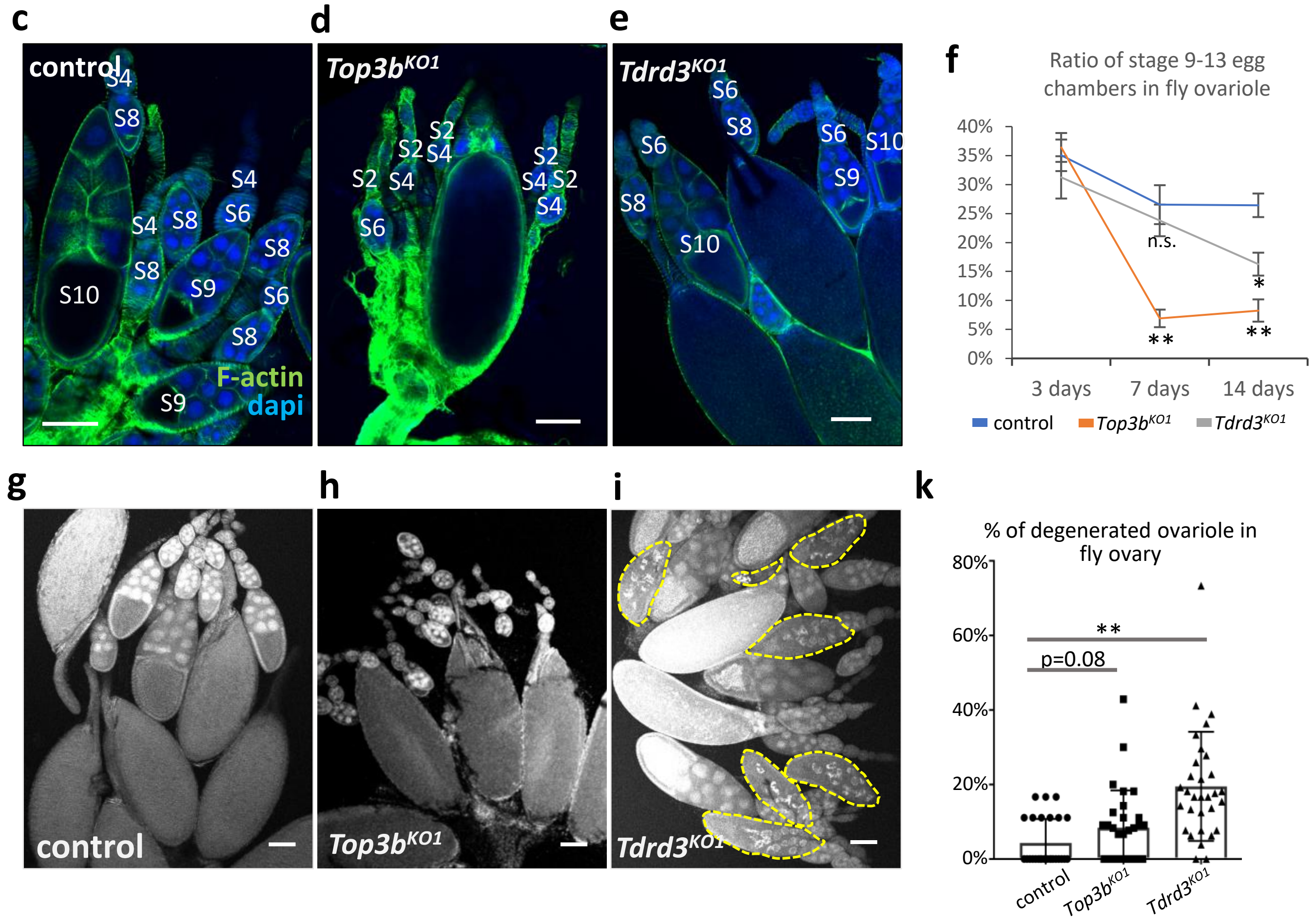

Figure S7. continued

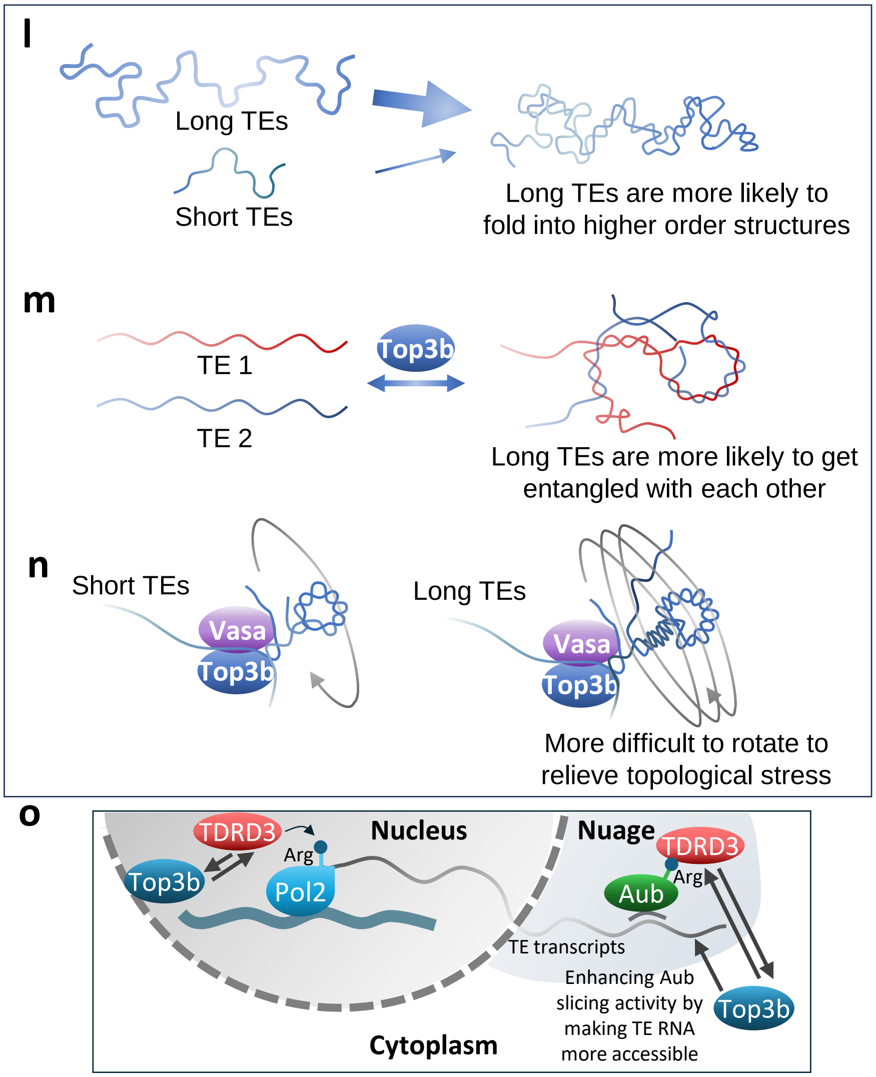

Figure S7. Top3b-Tdrd3 promotes fertility in aging associated manner in mouse and flies, and models how Top3b promotes piRNA driven TE silencing (a) Fertility test of *Tdrd3-KO* mouse. Total pup number and delivery frequency were counted in (b). There were total 4 pairs for each cross (n=4). The experiment was monitored for 8 months. (b) Average pup number per delivery was calculated from the pup counting. (c-f) Confocal images of 14 days old ovarioles stained with phalloidin and dapi and quantification (f). Note that *Top3b* mutant (d) exhibits stalling of development at stage 4-6 whereas control (c) and *Tdrd3* mutant (e) display gradual progress of development. (g-k) Representative confocal images with dapi staining showing degenerative egg chamber in *Tdrd3*^{KO1} mutant (i) as compared to control (g) and *Top3b*^{KO1} mutant (h). (k) quantification of degenerative phenotype. Data are represented as mean +/- SEM. Statistical analysis was done by two-tailed t-test (*:$p<0.05$).(l) Long and highly expressed TE RNAs may be easier and more likely to fold into complex and higher order topological structures (such as knots) than short and lowly expressed ones. (m) Longer and highly expressed TE RNAs may be easier and more likely get entangled with each other, which may cause topological problems and require topoisomerase to untangle them. (n) Longer and highly-expressed TE RNAs may be more difficult to release topological stress by rotation than shorter and lowly-expressed ones. Topological stress, such as supercoils, may be produced by helicases (such as Vasa) during unwinding of duplex RNA regions. Top3b may resolve such topological structures during PTGS and piRNA biogenesis. (o) A model to explain how TDRD3 but not Top3b is enriched in Nuage through an aub-dependent mechanism. We hypothesize that TDRD3 is recruited to Nuage by Aub through interactions between methylated arginine residues in Aub and Tudor domain of TDRD3. This is the mechanism of how Aub recruits other Tudor proteins to Nuage. We also hypothesize that the interaction between Top3b and Aub-bound TDRD3 is transient, so that Top3b localization in Nuage is also transient, unlike that of TDRD3. Once in a complex with TDRD3 and aub, Top3b can enhance Aub slicing activity by untangling higher TE RNA structures, and make them more accessible for Aub to slice. Related to Figure 7.
